# Spatio‐temporal dynamics of exotic fish species in the Mediterranean Sea: Over a century of invasion reconstructed

**DOI:** 10.1111/gcb.16362

**Published:** 2022-09-02

**Authors:** Ernesto Azzurro, Sonia Smeraldo, Manuela D'Amen

**Affiliations:** ^1^ IRBIM CNR Institute of Biological Resources and Marine Biotechnologies – National Research Council Ancona Italy; ^2^ Zoologica Station Anton Dohrn Naples Italy; ^3^ Istituto Zooprofilattico Sperimentale del Mezzogiorno Naples Italy; ^4^ The Italian Institute for Environmental Protection and Research ‐ ISPRA (PRES‐PSMA) Rome Italy

**Keywords:** biotic homogenization, fish invasion, historical trends, Lessepsian invasion, Mediterranean, spread rate

## Abstract

With over a thousand of introduced species, the Mediterranean is the most heavily invaded marine region in the world. Yet, the spatio‐temporal dynamics of this bioinvasion has never been analysed. Examination of a comprehensive dataset of 4015 georeferenced observations, extracted from the scientific literature, allowed (i) reconstructing the invasion and the introduction and post‐introduction dynamics of exotic fish species, (ii) calculating introduction and spread rates, and (iii) investigating the time correlates since introduction. Our analysis encompasses 188 fish species that entered the Mediterranean from 1896 to 2020, including 25 Atlantic species that naturally expanded their range through the Strait of Gibraltar. Cumulative occurrences, reported in 264 distribution maps, documented the progressive expansion of the most represented species and the spatio‐temporal patterns associated with three introduction routes: the Suez Canal (CAN); other human‐mediated vectors (HM) and the Strait of Gibraltar (NRE). The arrival rate of the species introduced through all three routes increased steeply after 1990, without a sign of saturation. Data analysis highlighted some temporal and geographical patterns, such as the effect and eventual weakening of the biogeographical barriers represented by the Strait of Sicily and the North Aegean Sea and the asymmetrical distribution of occurrences along the northern and southern Mediterranean coasts. Finally, there was an exponential increase in the secondary spread rates of CAN and NRE immigrants, as the more recent introductions achieved the fastest geographical expansions. Our findings provide a detailed and spatially explicit summary of a massive invasion that has changed the history of the Mediterranean biota and represent a remarkable example of rapid biotic homogenization in the global ocean.

## Graphical abstract

Between 1896 and 2020, a total of 188 fish species entered the Mediterranean Sea, mainly from the Suez Canal, but also from other human‐mediated vectors and from the Straits of Gibraltar. Cumulative occurrences, reported in 264 distribution maps, documented this phenomenon, which increased steeply after 1990, without a sign of saturation. The more recent introductions achieved the fastest and most spectacular geographical expansions. Our study provides a spatially explicit summary of a massive invasion that has changed the history of the Mediterranean biota.

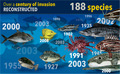

## 1 INTRODUCTION

Marine bioinvasions are a centuries‐old phenomenon (Carlton, 1996; Ojaveer et al., 2018). However, in the past few decades human activities have dramatically accelerated their pace all over the world (Carlton, 1996; Fowler et al., 2020). Like other global environmental issues, the spatial and temporal extent of marine bioinvasions is subject to increasing observational efforts (Sagarin & Pauchard, 2010), especially in areas like the Mediterranean Sea, which is a major invasion hotspot (Edelist et al., 2013). This basin, which is naturally rich in species and endemisms (Coll et al., 2010; Lejeusne et al., 2010), is undergoing a climate‐related decline of the native biodiversity (Albano et al., 2021) and a continuous invasion of exotic species. More than 1000 non‐indigenous taxa have been detected to date (Zenetos & Galanidi, 2020), including rising numbers of exotic fish (Azzurro et al., 2022; Golani et al., 2021). The most important entry route is the Suez Canal. Since its opening in 1869, hundreds of species from the Red Sea (Galil et al., 2017; Zenetos et al., 2017) have entered the Mediterranean via this artificial route. Today, such ‘Lessepsian immigrants’ account for more than a hundred fish species (Golani et al., 2021). Several of these organisms have established large, permanent populations in the eastern Mediterranean and are spreading westwards, causing a variety of ecological and socioeconomic impacts (Katsanevakis et al., 2014). Other species have entered through other human vectors, particularly shipping and the aquarium trade (Zenetos & Galanidi, 2020). Finally, the Strait of Gibraltar enables the active entry of Atlantic fish into the Mediterranean. This natural opening has been contributing to the composition of the Mediterranean marine fauna and flora since geological times and, according to the most widely accepted definitions (e.g., Carlton, 1996; Olenin et al., 2010), species entering through this route without direct human assistance (‘newcomers’ sensu Evans et al., 2020 or ‘neonatives’ sensu Essl et al., 2019) can be considered neither as true aliens nor as non‐indigenous species. Nevertheless, in some remarkable cases, they are listed together with ‘true’ exotic species (Golani et al., 2021). Regardless of their origin, a knowledge of the spatio‐temporal dynamics of the invasion of these three groups of species would be useful to assess the rapid and irreversible transformation of the Mediterranean ichthyofauna, which some authors have called ‘demediterraneization’ (Quignard & Tomasini, 2000) and others ‘tropicalization’ (with reference to invasive species of tropical origin; Bianchi & Morri, 2003). In the past few decades, the two processes have shown a rapid acceleration (Golani et al., 2021), due to increasing trade volumes (Flagella et al., 2007; Gollasch & David, 2019) and to warmer and saltier Mediterranean waters (Azzurro et al., 2019; D'Amen & Azzurro, 2020a; Lasram et al., 2008; Marras et al., 2015). The spatio‐temporal spread dynamics may vary widely in relation to species and entry route; however, irrespective of the route, some species develop successful populations and spread over large geographical areas (e.g. Golani et al., 2021; Karachle et al., 2004), whereas others may remain rare for long periods before spreading, or even fail to establish permanent populations (Azzurro et al., 2014, 2016; Golani et al., 2021).

There is now a considerable body of data on the occurrence on exotic fish species (sensu Golani et al., 2021) in the Mediterranean, whose most likely introduction routes have recently been reviewed by Golani et al. (2021). Fish are involved in several of the most emblematic invasions in the global ocean (Edelist et al., 2012). Moreover, compared with other exotic organisms, they are conspicuous, easily recognized and they interact with human activities, chiefly fisheries. This creates relatively favourable conditions for detection and for collecting occurrence data, which provide key information for the study of biodiversity changes (Petersen et al., 2021).

We exploited a new, large compilation of georeferenced occurrences that have recently been extracted from the Mediterranean literature through the ORMEF (Occurrence Records of Mediterranean Exotic Fishes) database (Azzurro et al., 2022). The dataset—which consists of 4015 georeferenced observations of 188 fish species new to the Mediterranean Sea—offers an invaluable opportunity to explore a centuries‐old invasion across space and time in a consistent way over an entire marine region. In this study, we drew time‐based cumulative occurrence maps; calculated accumulation curves of the new species and related occurrences; examined their progressive expansion and the resulting distribution along the longitudinal axis of the basin; estimated species spread rates and tested the relevant time correlates since their introduction in relation to a set of four critical variables.

## 2 METHODS

### 2.1 Species selection and georeferenced records

Our study is based on the georeferenced ORMEF database (Azzurro et al., 2022). Earlier versions have been employed for large‐scale investigations of invasive species (e.g. Azzurro & D'Amen, 2022; D'Amen & Azzurro, 2020a, 2020b; Parravicini et al., 2015). The current version comprises the occurrence records of presumably all the non‐indigenous fish species recorded in the Mediterranean to date, including recent Atlantic immigrants.

We considered three broad groups of species based on their introduction route (Golani et al., 2021), as follows: CAN = exotic fish of Red Sea origin, introduced through the Suez Canal; HM = exotic fish introduced by other human‐mediated vectors, such as shipping, mariculture and aquarium release; and NRE = fish of Atlantic origin naturally spreading through the Strait of Gibraltar. Although the latter group does not fall into the most widely accepted definition of non‐indigenous species (Carlton, 1996; Olenin et al., 2010), in line with previous Mediterranean inventories (e.g. Golani et al., 2021) their occurrences in the basin deserve tracing and can be used in appropriate comparisons (Lasram et al., 2008). We analysed 188 fish species and 4015 georeferenced observations spanning from the first record of *Pampus argenteus* in 1896 to the latest recorded occurrence of *Cheilodipterus novemstriatus* in August 2020. In line with Golani et al. (2021), we distinguished between species that established and did not establish permanent populations in the invaded range. Only records identified at the species level were included, whereas genus‐level identifications (e.g. *Abudefduf* spp. in Dragičević et al., 2019) were not considered. Altogether, there were 106 CAN (3707 observations), 57 HM (113 observations) and 25 NRE (195 observations) species. These data were used to reconstruct the spatio‐temporal dynamics of Mediterranean fish invasion in the last 125 years.

### 2.2 Cumulative occurrence maps

Georeferenced observations were converted to a World Geodetic System 1984 datum and imported into ArcGIS desktop version 10.2 (ArcGIS ESRI, 1999). We mapped the cumulative occurrences of CAN, HM and NRE species from 1900 to 2020 at intervals of 30 years until 1960 and of 10 years until 2020, obtaining nine cumulative maps of occurrence records. For the 26 species with at least 20 georeferenced records in the database, we realized cumulative occurrence maps at 10‐year intervals, starting from 1930, that is 10 maps per species (Appendix S1; Figures S1–S26). We assumed that the map gathering all records represents the distribution of each species at the time of the study (until August 2020).

### 2.3 Species accumulation and species observations

The cumulative number of species' first records collected over 125 years was plotted for each entry route (CAN, HM and NRE) and according to both, established‐species only and all species. To rule out potential artefacts due to changes in research efforts, we also calculated the cumulative number of sightings. A breakpoint structural analysis was performed to assess the year(s) of statistically significant change(s) in the accumulation rates. We also tested the correlation of the number of records with the total number of species.

Cumulative curves of species introductions were calculated for the CAN and NRE routes along the longitudinal Mediterranean axis and a breakpoint analysis was applied to highlight any biogeographical breaks in their spatial distribution. For each category, the time series were randomly split into two or more subsets and the mean level was compared using a modified *F* test (‘structural change’ or sc test, Zeileis et al., 2003). The procedure is repeated iteratively until all significant breakpoints (if any) are identified (Bai, 1994). The Bayesian information criterion was applied as an objective criterion to determine the breakpoint number. For breakpoint analysis, we used the ‘strucchange’ package in R 4.0.2 software (R Core Team, 2020).

### 2.4 Spread rates

Spread rates from the entry point were calculated for CAN (Port Said) and NRE species (Strait of Gibraltar), but not for HM species, because in their case preliminary data exploration did not highlight a definite direction of geographical spread. To provide a realistic reconstruction of the advance of CAN and NRE species, data points along the southern Mediterranean coast (from the Suez Canal opening to Gibraltar along the African coast) were analysed separately from those located along the northern Mediterranean coast (from the Suez Canal opening to Gibraltar along the Asian and European coasts). We only considered species with a number of records (>10) that would enable reliable quantification of dispersal, namely distance from the entry point. For each species (38 CAN and 5 NRE), we calculated the distance between consecutive records by two approaches: least‐cost distance spread in the sea along the Mediterranean coast (in km) and maximum longitudinal distance covered in successive time lapses (in decimal degrees).

The former approach allowed calculating the distance of consecutive records at an increasing distance from the Suez Canal, where possible confining the movement to the continental shelf without crossing land (D'Amen & Azzurro, 2020a; Hiddink et al., 2012). To do this, we used the function shortestPath in the ‘gdistance’ package (Van Etten, 2017) in R 4.0.2 software, which allows calculating the least‐cost distance between point pairs. We created a conductance layer that measured the local ‘friction’ of the landscape by assigning the highest permeability to the continental shelf area and declining permeability values to areas with increasing depth. We used distance (km) and time information (years) to calculate the spread rates of the dispersal progress, that is, the spread rate with each farther record from the Suez Canal (CAN) and the Strait of Gibraltar (NRE). Finally, for each species, we calculated an average spread rate from the entry site to the most distant record.

The second method measures the cumulative distance covered by each species along the longitudinal axis since the year of first detection, based on the same chronological series as the dispersion progress. In this case, we computed in ArcView 10.2, the linear distance between chronologically consecutive records along the longitudinal axis for the northern and the southern coast. Since the spread rates obtained with the two different approaches showed a strong correlation in both coasts (*ρ* > .89, Spearman correlation test; *p* < .0001), for the subsequent analyses, we used only the results of the former method. The results of maximum linear longitudinal distance analysis are reported in Appendix S5.

### 2.5 Factors correlated with the time since introduction

The possible influence of the time since introduction, expressed as ‘minimum residence time’ (sensu Simberloff & Rejmanek, 2010), on species spatio‐temporal dynamics, was investigated only in established species, by plotting the year of the first record against four variables: maximum expansion along the longitudinal axis; number of species records; and mean spread rates (km year^−1^) along the northern and the southern coast.

## 3 RESULTS

### 3.1 Mapping introduction and post‐introduction histories

The georeferenced records provided by the ORMEF database allowed mapping the long‐term accumulation of new fish species that entered the Mediterranean since 1869.

**FIGURE 1:**
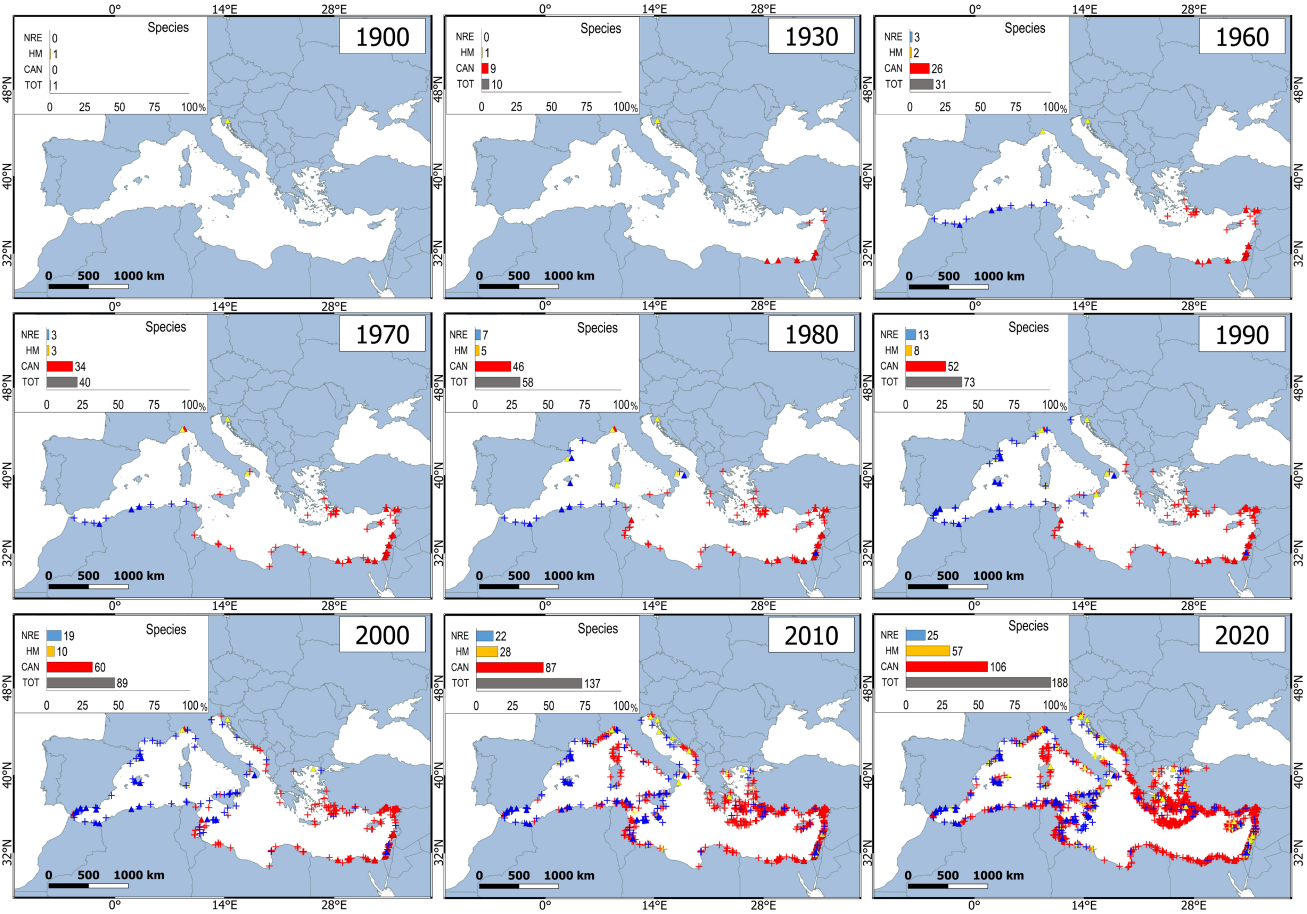
Cumulative occurrences of fish species that entered the Mediterranean: Red symbols = Lessepsian species introduced through the Suez Canal (CAN); Yellow symbols = human‐mediated introductions through shipping, aqua/mariculture, aquarium release etc (HM); blue symbols = expansion of the natural range through the Strait of Gibraltar (NRE). Triangles = first records. Crosses = further records. Nine‐time intervals from 1900 to 2020 are displayed. The cumulative percentage of species and records is shown in each map. Map lines delineate study areas and do not necessarily depict accepted national boundaries.

Until 1930, the records regarded only a small number of exotic species, that are, *Pampus argenteus* (HM) and nine Lessepsian immigrants (CAN species): *Alepes djedaba*, *Atherinomorus forksali*, *Coryogalops ochetica*, *Crenidens crenidens*, *Equulites klunzingeri*, *Hemiramphus far*, *Chelon carinatus*, *Siganus rivulatus* and *Stephanolepis diaspros* (Figure 1). The first documented observation of an NRE fish regarded *Psenses pellucidus* in 1955. After 1930, a rising number of species and observations accumulated for all the categories (Figure 1; Appendix S1). Until the 1990s, the distribution of Lessepsian fish was mostly confined to the easternmost sectors of the Mediterranean. Notably, before 1990, only two CAN species had reached the western Mediterranean, respectively Italy (*Pomadasys stridens* in 1968) and Tunisia (*Siganus luridus* in 1969). After 1990, their geographical expansion was massive in terms of both number of records and newly occupied areas, with several species extending their range to the western and northern Mediterranean sectors and the eastern Adriatic Sea (Figure 1).

The Atlantic species (NRE) moved in the opposite direction. By 2010, six of them had reached the eastern Mediterranean. A rising number of NRE species was also recorded throughout the basin, though with a limited number of observations per species. Finally, species introduced by other human activities (HM), such as shipping, aqua/mariculture and aquarium release, showed scattered isolated records lacking a clear geographical direction or apparent spread. These species were generally represented by one or two records (31 and 14 species, respectively), 13 records being achieved only by *Cephalopholis taeniops*. Again, sightings greatly increased after 1990. Individual cumulative occurrence maps are provided for 26 fish, 23 CAN and 3 NRE species, for which more than 20 records are available in 10 decades (Appendix S1).

### 3.2 Accumulation curves

**FIGURE 2:**
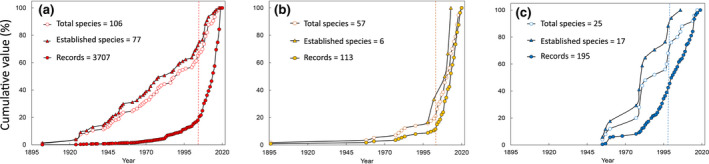
Temporal accumulation trends of exotic fish and records, according to the three entry routes: (a) CAN = Suez Canal; (b) HM = human‐mediated transport; (c) NRE = natural range expansion through Gibraltar. Absolute numbers are shown in the upper left corner of each graph. The vertical dashed line after 1990 marks the year when the accumulation of records of each group exhibited a significant structural change.

Historical occurrence records allowed close tracking of the progressive accumulation of exotic fish introduced through the CAN, HM and NRE routes (Figure 2a–c). Data analysis yielded very different outcomes, with CAN species representing by far the major group both in terms of species detected and of records (106 and 3707, respectively). Cumulative curves (Figure 2) were drawn to illustrate the differences and commonalities of the different entry routes. In all groups, accumulation was always non‐linear and best described by exponential functions. For all routes, their cumulative number (both all species and established species alone) significantly correlated with the cumulative number of sightings (*p* < .001), with Spearman's *ρ* values of .85, .99 and .96 for all CAN, HM and NRE species, respectively, and .80, .99 and .95 for established CAN, HM and NRE species.

Sightings showed a sudden increase in the 1990s, with significant breakpoints in 2002, 2003 and 1997 for CAN, HM and NRE species, respectively. Notably, over 90% of sightings occurred after 1990, as the rate of detected arrivals jumped from 0.6, 0.2 and 0.2 species year^−1^ before 1990 to 2.3, 1.7 and 0.4 species year^−1^ after 1990 for CAN, HM and NR species, respectively.

### 3.3 Spatial trends

The geographical progress of CAN and NRE invasions was well represented along the longitudinal axis, with maximum range expansions being recorded for CAN species. Three of these, *Fistularia commersonii*, *Lagocephalus sceleratus* and *Etrumeus golani*, are recorded up to the westernmost sectors of the Mediterranean, at the maximum possible distance from their entry point at Port Said (Appendix S1). Among established species, 17 (16%) CAN and 7 (28%) NRE species successfully crossed the Strait of Sicily to occupy both the eastern and western sectors of the Mediterranean.

**FIGURE 3:**
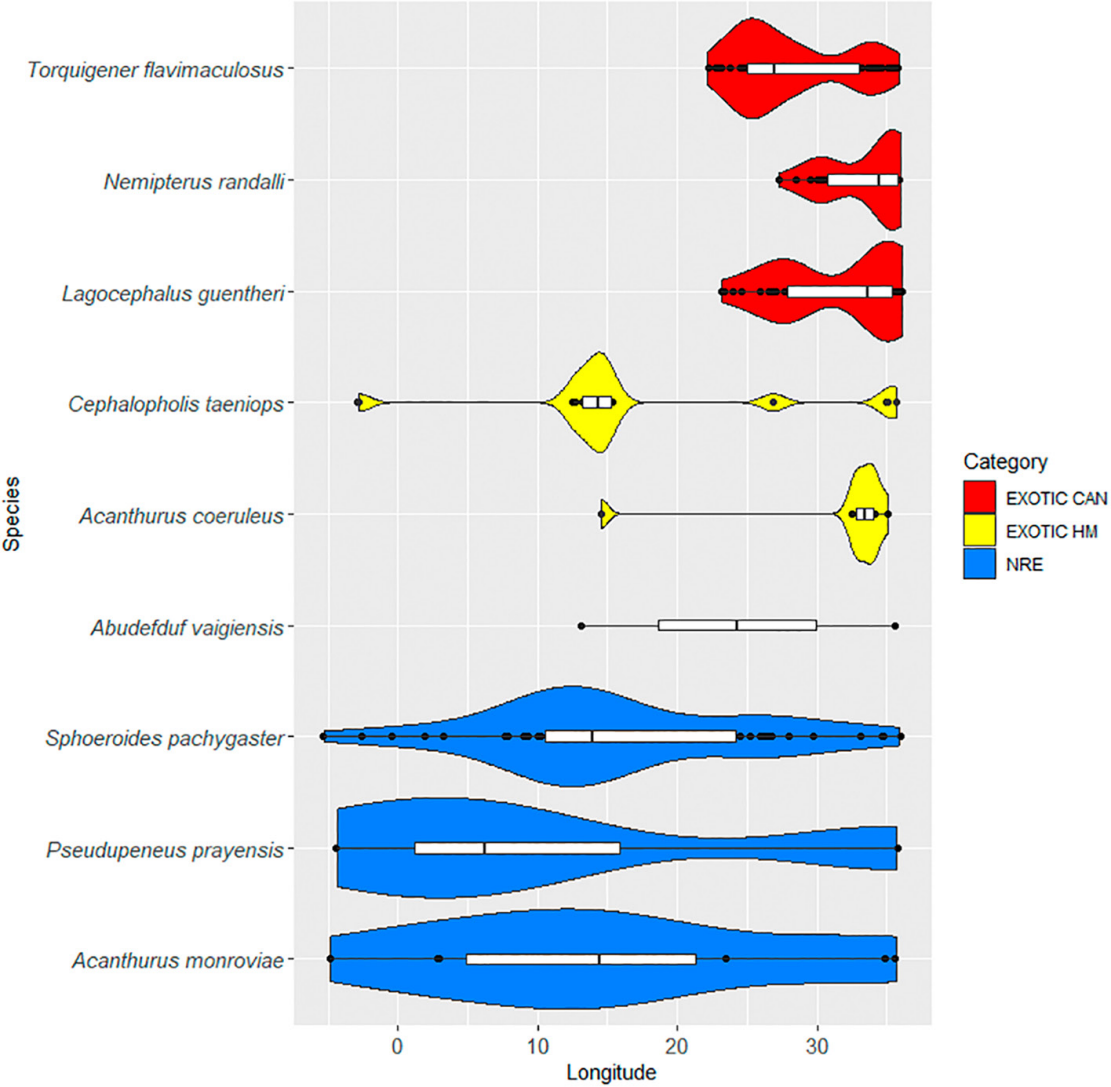
Violin plots of 9 species, 3 per route, showing how the data records of fish introduced through the CAN, HM and NRE routes can vary along longitude, using density curves based on kernel probability. The boxplot summarizes the centre and spread of the distribution: the box centre represents the median and its length the interquartile range. Black dots: records outside the interquartile. The violin plots of the other species are reported in Appendix S3.

Violin plots (Figure 3) showed different spatial distribution patterns for the three groups of species along the longitudinal axis. NRE and HM are mostly represented by few records dispersed over wide distances; for example, the West African goatfish *Pseudupeneus prayensis* (NRE) was recorded only four times in the Mediterranean, but along a geographical range spanning from Lebanon to Tunisia. In contrast, CAN species are characterized by much denser spatial aggregations that accumulate in the eastern sectors (see Appendices S2–S4). Notably, the records of CAN species along the longitudinal axis rapidly diminish in correspondence of the Aegean Sea and the Strait of Sicily, becoming much less frequent in the western Mediterranean (see Figure 3; Appendices S2 and S3). This observation was confirmed by breakpoint analysis of the cumulative number of species, which identified two main breaks at 28.50 and 11.06 decimal degrees of longitude, for the Aegean Sea and the Strait of Sicily, respectively (Appendix S3). Likewise, the accumulation curves for NRE species, fell markedly in correspondence of the Strait of Sicily. The graphical evidence is confirmed by the significant breaks detected at 13.00 decimal degrees of longitude (Appendix S3). Finally, no clear patterns were visible for HM species, whose distribution is represented by a limited number of records scattered along the longitudinal axis, without any clear sign of a geographical expansion.

### 3.4 Spread rates

Spread rates along the northern and southern coasts of the Mediterranean were calculated by the least‐cost method for species with more than 10 occurrences. HM species were excluded because they showed no clear expansion direction. Altogether, this analysis involved 43 CAN and 4 NRE species along the northern coast and 30 CAN and 5 NRE species along the southern coast. Notably, 13 CAN species, largely distributed along the northern coast, have not yet been reported in the southern coast, except for one, which has been sighted once in Egypt.

The detailed spread rates, reported in Appendix S5, are expressed in km year^−1^. The respective median, minimum and maximum values along the northern (N) and the southern (S) coast were: CAN_N_ = 105.98, 20.9, 705.4; CAN_S_ = 76.20, 14.91, 335.21; NRE_N_ = 36.69, 15.18, 360.83; and NRE_S_ = 180.24, 53.89, 499.93. The spread rates of CAN species along the northern and southern coasts were not significantly different (Wilcoxon paired test, *p* > .1). The silver‐cheeked toadfish *Lagocephalus sceleratus* (CAN) and *Sphoeroides pachygaster* (NRE) showed the fastest dispersal. Spread rates positively correlated with the total species dispersal only along the southern coast (Spearman correlation test, *ρ* = .701, *p* < .0001), whereas no significant correlations were found for the northern coast. Interestingly, the 15 CAN species that successfully spread beyond the Strait of Sicily were characterized by significantly higher spread rates than the other species (Wilcoxon test, *V* = 2080, *p* < .0001).

### 3.5 Correlates of time since introduction

Investigation of the correlation between the year of first record and four key variables—the maximum expansion along the longitudinal axis; the number of species records; and the mean spread rates along the northern and southern coasts—highlighted a significant negative correlation with the maximum longitudinal expansion of CAN species (Spearman correlation test, *ρ* = −.49, *p* < .0001), which indicates that the earliest immigrants achieved a wider geographical expansion. This pattern was also apparent, albeit not significant (*ρ* = −.42, *p* > .05) for NRE species. A weaker, but significant negative correlation was found with the total number of records of CAN species (*ρ* = −.32, *p* < .05).

**FIGURE 4:**
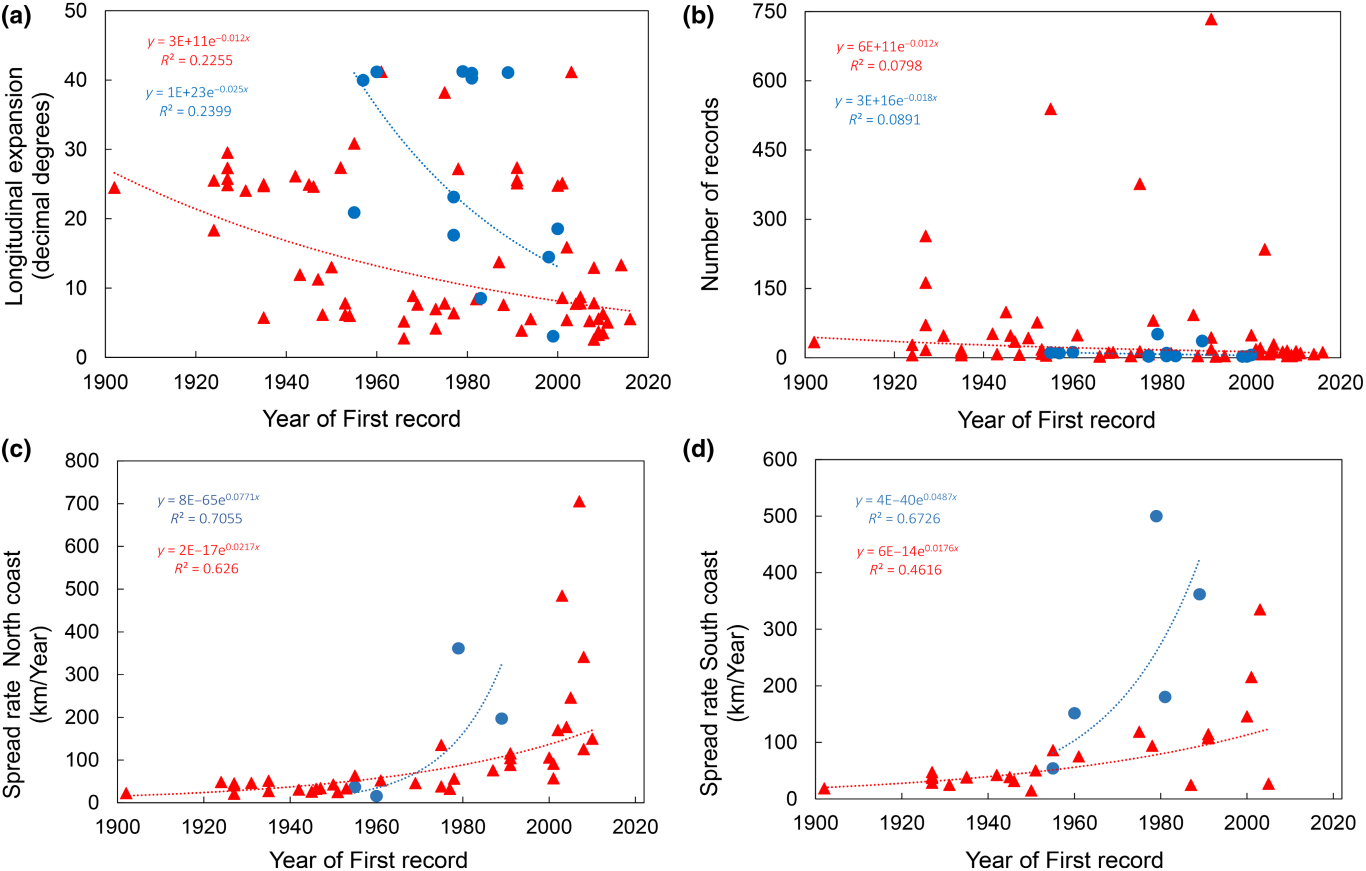
The year of first Mediterranean record plotted against: maximum expansion along the longitudinal axis (a); number of species records (b); and mean spread rates (km/year) along the northern (c) and southern (d) coasts. Red triangles: CAN; blue circles: NRE. The trend lines are shown and the resulting expressions are reported in the plot.

The year of first record showed positive correlations with the spread rates of CAN species along both coasts (Spearman correlation test: CAN_N,_ *ρ* = .81, *p* < .0001; CAN_S_, *ρ* = .59, *p* < .05), providing sound evidence that the more recent arrivals spread faster than the earlier immigrants. Notably, along the northern coast, all the CAN species that entered the Mediterranean before 1990 shared comparable spread rates (44.05 ± 25.3 km year^−1^), whereas those that were introduced after 1990 showed a significantly greater average speed (Figure 4c; Appendix S4). Higher average spread rates were also apparent for the southern coast, but with a less marked difference, since a fast spread was already recorded after 1960 (Figure 4d).

For CAN species, the year of first record was significantly fitted (*p* < .05) by an exponential regression curve against the four variables (Figure 4).

## 4 DISCUSSION

Species invasions are rarely described from their very beginning (Pyšek & Hulme, 2005) and can be highly difficult to track in the marine environment (Carlton, 1996). Today, the increasing diffusion of social media, participatory practices and open data policies has considerably amplified our observation capabilities (Katsanevakis et al., 2020; Schade et al., 2019). However, georeferenced information on spatio‐temporal dynamics is seldom found in the scientific literature and is not yet available from public databases.

In this study, we provide a synthesis of the invasions of fish—one of the most closely monitored groups of marine organisms—by exploiting the unique situation of the Mediterranean Sea as a world's hotspot for biological invasions. Occurrence records published by hundreds of scientists in the past 120 years (Azzurro et al., 2022) allowed reconstructing the spectacular history of a massive invasion. Few similar efforts (Pyšek & Hulme, 2005; Ricciardi, 2006, 2016) are found in the global literature.

### 4.1 Mapping introduction and post‐introduction histories

With 269 species occurrence maps, we documented the spatio‐temporal progression of the invasion of the Mediterranean according to the three introduction routes, CAN, HM and NRE. Since all else being equal, the faster the spreads of an invading species, the more dangerous the invasion (Essl et al., 2019), such maps are particularly useful to inform stakeholders, policymakers and the general public in numerous geographical regions of the actual scope of the problem (Hawthorne et al., 2015). Moreover, tracking non‐indigenous species provided comprehensive and explicit information for the EU 2030 biodiversity strategy and for regional initiatives such as the Marine Strategy Framework Directive (EU, 2017) and the Integrated Monitoring and Assessment Programme (IMAP) and related Assessment Criteria, adopted by the Contracting Parties to the Barcelona Convention (IMAP, 2017). The occurrence maps, explored in terms of species or route, provide sound baseline data for risk assessment and are easily updated as the invasions advance.

### 4.2 Temporal trends of introduction

Introduction trends provide simple but essential information (Seebens et al., 2021) for national (Zenetos et al., 2020) and regional (Galil, 2009) inventories, notably because the number of introduced species, combined with the available published sightings allows exploring the process in its evolution and observation.

The increasing rates of introduction, seen for all three groups, can be ascribed to the greater effectiveness of the prevalent routes and vectors (Galil, 2007; Zieritz et al., 2017). The Suez Canal became more permeable after the completion of the Aswan High Dam in 1964, the dilution of bitter lakes and the expansion of its cross‐sectional area, with the recent excavation of a second canal (Biton, 2020; Galil et al., 2015). In contrast, the growing number of Atlantic species has been ascribed by some authors (e.g. Evans et al., 2020) to changes in water circulation patterns and/or to more suitable environmental conditions at Gibraltar.

The steep rise in the cumulative number of sightings of all three species groups in the 1990s demonstrates a greatly increased observation capacity in the past few decades. This finding mirrors the general expansion of research into alien species—which has also risen significantly in the 1990s (Richardson & Pyšek, 2008; Thomaz et al., 2015)—and the recent upsurge of citizen‐generated observations, which have provided a large amount of new records to the Mediterranean literature (Katsanevakis et al., 2020; Zenetos et al., 2020). Such greater observational capacity is particularly useful for the detection of rare or not established species (e.g. Tiralongo et al., 2019). This is clearly the case of fish transported by ships, released by aquaria and introduced via other human‐mediated activities, as shown by the cumulative curves of HM species, which closely mirror record accumulation.

For all three routes considered in the study, species introductions follow exponential trends that show no signs of slowing down, thus providing a new and remarkable example of unsaturated trends of invasion (Ricciardi, 2016; Ruiz et al., 2011; Seebens et al., 2021). It has been highlighted that earlier Lessepsian and Atlantic immigrants are generally more abundant than the later arrivals (Golani et al., 2021). This means that the large number of species introduced in the past few decades have yet to produce their full impact (‘invasion debt’; Essl et al., 2011; Galil et al., 2021), and that more exotic species and more successful invasions should be expected in the near future.

### 4.3 Spatial trends

The distribution of an exotic species reflects its entire invasion process from introduction to establishment and subsequent spread (Murray et al., 2014). Whereas, HM species are mostly represented by isolated records, without clear signs of a geographical expansion, we were able to track the advance of several CAN and NRE species from their respective entry points at Port Said and Gibraltar. Interestingly, their expansion slowed down in correspondence of the Strait of Sicily and the North Aegean Sea, were important biogeographical and climatic transitions occur (D'Amen & Azzurro, 2020a). These data (Appendix S4) agree with the observation of a slower dispersal of Lessepsian fish in these sectors (Lasram et al., 2008), which have long been held to act as filters to their western spread (Por, 1990; Quignard & Tomasini, 2000). We must however consider that today 15 Lessepsian fish have already passed through them.

We also found a different distribution of CAN species in the northern and southern coasts. Remarkably, while all CAN species are found along the northern coast, only a minority are detected along the southern coast. This is the case of *Apogonichthyoides pharaonis*, *Bregmaceros nectabanus*, *Equulites klunzingeri* and of 37 other Lessepsian fish. This asymmetrical distribution, previously described by Mavruk and Avsar (2008), cannot be attributed to climate conditions (D'Amen & Azzurro, 2020a) but appears to be primarily related to the Nile freshwater inputs, which create a saline barrier to the westward spread, and possibly to the counterclockwise water circulation in the eastern Mediterranean (Pinardi & Masetti, 2000), which may prevent the estward drift of eggs and larvae.

The spatio‐temporal dynamics of exotic species is critical for our understanding of biodiversity patterns (Seebens et al., 2021) and should be considered in relation to the ongoing environmental changes affecting the Mediterranean Sea. The negative correlation between the year of first record and maximum longitudinal expansion suggests that the distribution of exotic species, especially Lessepsian immigrants, will continue to expand under the current, more favourable climatic conditions (D'Amen & Azzurro, 2020a). However, the fact that some of these species can occupy areas with temperatures lower than those experienced in their native ranges, and shift their niche accordingly (D'Amen & Azzurro, 2020b; D'Amen et al., 2022; Parravicini et al., 2015), raises the risk of underestimating their invasion success.

### 4.4 Spread rates

Least‐cost analysis, applied to the CAN and NRE species, allowed for the first time to quantify the spread rates of 43 Mediterranean fish. Such rates were generally higher than those of other taxa, shifting or introduced in other marine systems (Sorte et al., 2010), and were comparable to those calculated by other methods for some species with a lower number of records (Hiddink et al., 2012; Lasram et al., 2008). We assumed post‐establishment secondary spread to occur via natural dispersal. Analysis of the occurrence records along the southern and northern coasts of the Mediterranean found no significant difference in their speeds, suggesting a certain independence of the spread process of the main water circulation patterns, at least for the CAN and NRE species occurring along the southern and northern coasts.

Interestingly, CAN and NRE species showed a significant correlation between the year of first detection and the speed of their subsequent spread (Figure 4c,d). In other words, the more recent the introduction, the faster the expansion. This observation, which reinforces the hypothesis of a general acceleration of the invasion processes, may be explained by a weakening—due to climate change—of the climatic barriers that have previously prevented dispersal (Azzurro & D'Amen, 2022).

According to D'Amen et al. (2022), Lessepsian fish introduced after 1990 found temperature conditions analogous to those of their native ranges. This suggests a rapid expansion with a shortened lag phase, the delay in spread after the first introduction (Azzurro et al., 2016; Pyšek & Richardson, 2008). Other processes acting before the invasion, such as genetic changes prior to introduction (Chiesa et al., 2019), could also be involved in this recent increase of spread rates.

Since all else being equal, the faster the spreads of an invading species, the more dangerous the invasion (Essl et al., 2019), our findings provide further important evidence that highlights increasing invasion risk in the Mediterranean.

### 4.5 Possible biases

The reconstruction of the spatio‐temporal dynamics of Mediterranean fish invasions can be biased by the different ability to perceive it (Azzurro et al., 2016; Belmaker et al., 2009; Costello & Solow, 2003). For instance, the different research efforts of the different Mediterranean countries (Coll et al., 2010) are reflected in the uneven distribution of the occurrence records found in the ORMEF database (Azzurro et al., 2022). This factor can also bias the direct use of the discovery rate of exotic species as a robust estimate of their rate of introduction (Belmaker et al., 2009), especially for HM and NRE species, which show the highest correlation between number of species and number of records. However, since fish are relatively large, easy to detect and intensely interact with human activities (e.g. fishing and diving), the quantity and quality of the information presented here can be considered much higher and detailed than those available for other groups of exotic organisms.

The progressive expansion of the leading invasion edges was clearly traceable for most CAN species. Yet, another limitation of this work lies in the difficulty to reconstruct any clear sign of expansion for many NRE and HM species. In several cases, the wide spatio‐temporal distribution of their sightings over large distances would suggest a high speed of dispersion. Yet, the surmise was only substantiated by small numbers of recorded occurrences, without evidence of established populations. This, for example, is the case of *Pseudupeneus prayensis* whose only four total observations span from Tunisia to Lebanon; of *Oplegnathus fasciatus* first recorded in Malta and then only in the Gulf of Trieste; and of *Lutjanus sebae*, recorded merely twice, in Greece and in Sicily. These distribution patterns are unlikely to result from natural secondary long‐distance dispersal and suggest secondary, independent introductions. This pattern, which is seldom considered by the Mediterranean literature (Dimitriou et al., 2019), is common in aquatic bioinvasions (Simberloff & Rejmanek, 2010) and would entail the possibility that several NRE and HM species are not established at all in the sites where they have been recorded. Yet, this limitation is at the same time a significant result, since it raises the hypothesis of multiple introduction events, which deserve proper investigation by molecular studies (e.g. Frankham, 2005).

### 4.6 Species recorded after August 2020

This study is based on the ORMEF dataset, which was updated in August 2020 and has been enriched with about 500 new sightings of which 75% were published in 2021. This also includes records of *Oncorhynchus kisutch*, *Scolopsis ghanam* (Crocetta et al., 2021), *Orthopristis chrysoptera* (Tiralongo et al., 2020), *Pseudotolithus senegallus* (Akel, 2021), *Sargocentron spinosissimum* and *S. tiereoides* (Deef, 2021) and *Ambassis dussumieri* (Stern & Morov, 2022) detected for the first time in the Mediterranean.

Other sightings document range expansions of fish such as *Aluterus monoceros* (Crocetta et al., 2021) and *Rachycentron canadum* (Nour et al., 2021). In addition, *Pterois miles* and *Bregmaceros nectabanus* were reported for the first time in the mid‐eastern Adriatic Sea (Croatian waters) by Dragičević et al. (2021) and Orfanidis et al. (2021), respectively, who provided the northernmost record of the species in the Mediterranean Sea. Finally, *Fistularia petimba* was simultaneously recorded in different eastern Mediterranean areas (Crocetta et al., 2021), raising concern for a new successful invasion.

## 5 CONCLUSIONS

Our spatio‐temporal analyses describe a unique, massive and accelerating phenomenon of invasion in a marine region. Species continuously entering via different routes are progressively changing the faunistic identity of the Mediterranean basin, in a sort of *demediterraneization* of its biota, and with a growing *invasion debt* that is expected to produce escalating consequences. In the past few decades, exotic fish have become more numerous, more widespread and capable of much faster diffusion through the Mediterranean. Our findings summarize the invasion process and provide a benchmark against which future changes can be assessed.

## CONFLICT OF INTEREST

The authors declare no conflict of interest.

## FUNDING INFORMATION

This paper was supported by the Interreg‐Med Programme MPA‐Engage (1MED15_3.2_M2_337) and by the project @CNR USEIt.

## Supporting information


Appendix S1

Appendix S2

Appendix S3

Appendix S4

Appendix S5

## Data Availability

The data that support the findings of this study are available in SEANOE at https://doi.org/10.17882/84182 reference number Access on demand is requested up to 2022‐11‐05. After this date, data will be openly available.
