# Supplementary material for: Spatio‐temporal dynamics of exotic fish species in the Mediterranean Sea: Over a century of invasion reconstructed
Source: Glob Chang Biol. 2022 Sep 2;28(21):6268–79. doi: 10.1111/gcb.16362 (PMC9826093; doi:10.1111/gcb.16362)
Supplement: Supplementary file 1 — Appendix S1 [file GCB-28-6268-s001.pdf]

# SUPPLEMENTARY MATERIALS: Appendix 1

Spatio-temporal dynamics of the invasion in the Mediterranean Sea reconstructed by species for invasive fishes with more of 20 records (23 CAN, and 3 NRE species). Cumulative occurrences are shown in 10-year intervals from 1930 to 2020.

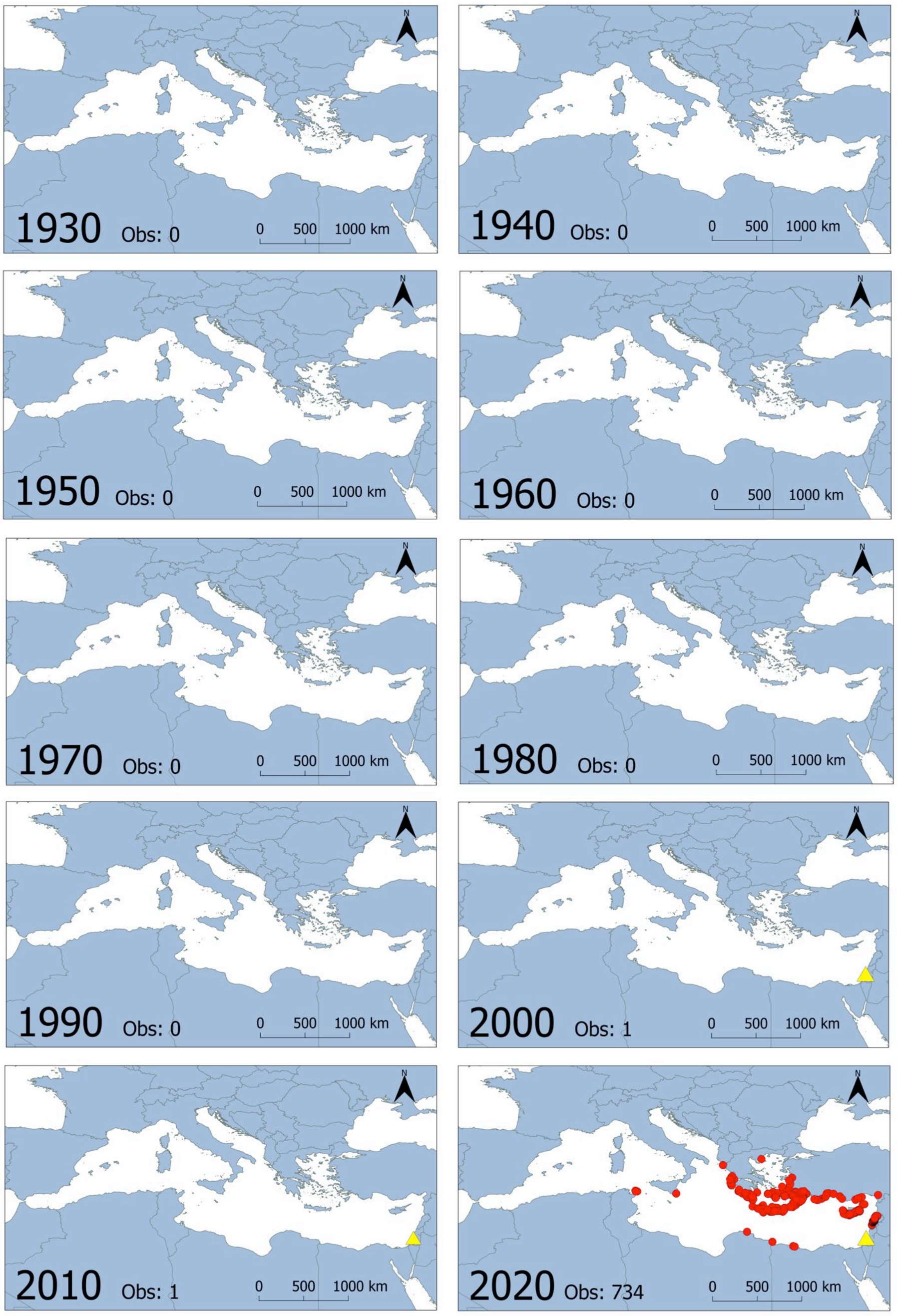


**Fig S1** Chronology of *Pterois miles* invasion in the Mediterranean Sea. **First record**: Israel, 1991. Cumulative occurrences are shown in 10-year intervals from 1930 to 2020.

Data consisted of 734 georeferenced records pooled from bibliographic sources.

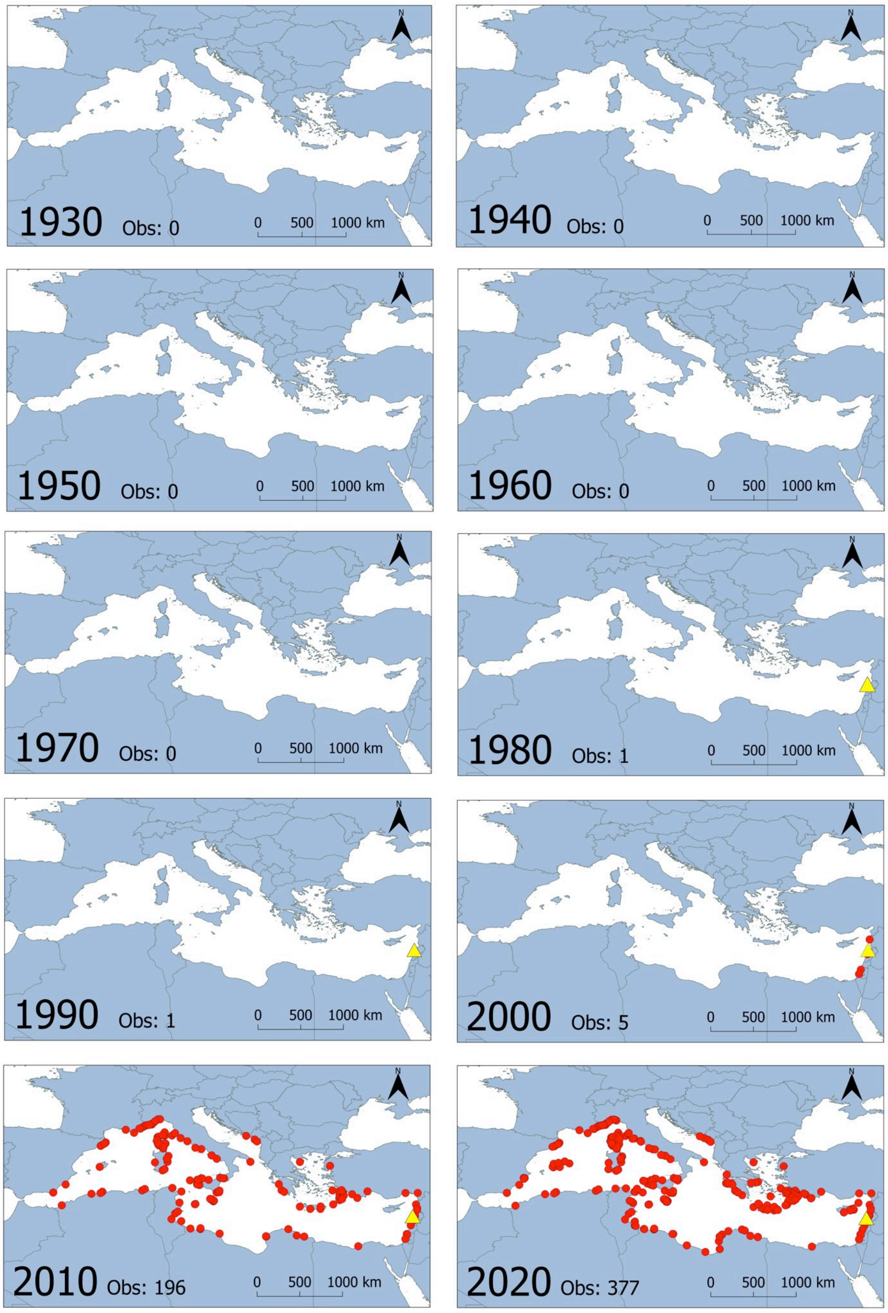


**Fig S2** Chronology of *Fistularia commersonii* invasion in the Mediterranean Sea. **First record**: Lebanon, 1975. Cumulative occurrences are shown in 10-year intervals from 1930 to 2020.
Data consisted of 377 georeferenced records pooled from bibliographic sources.

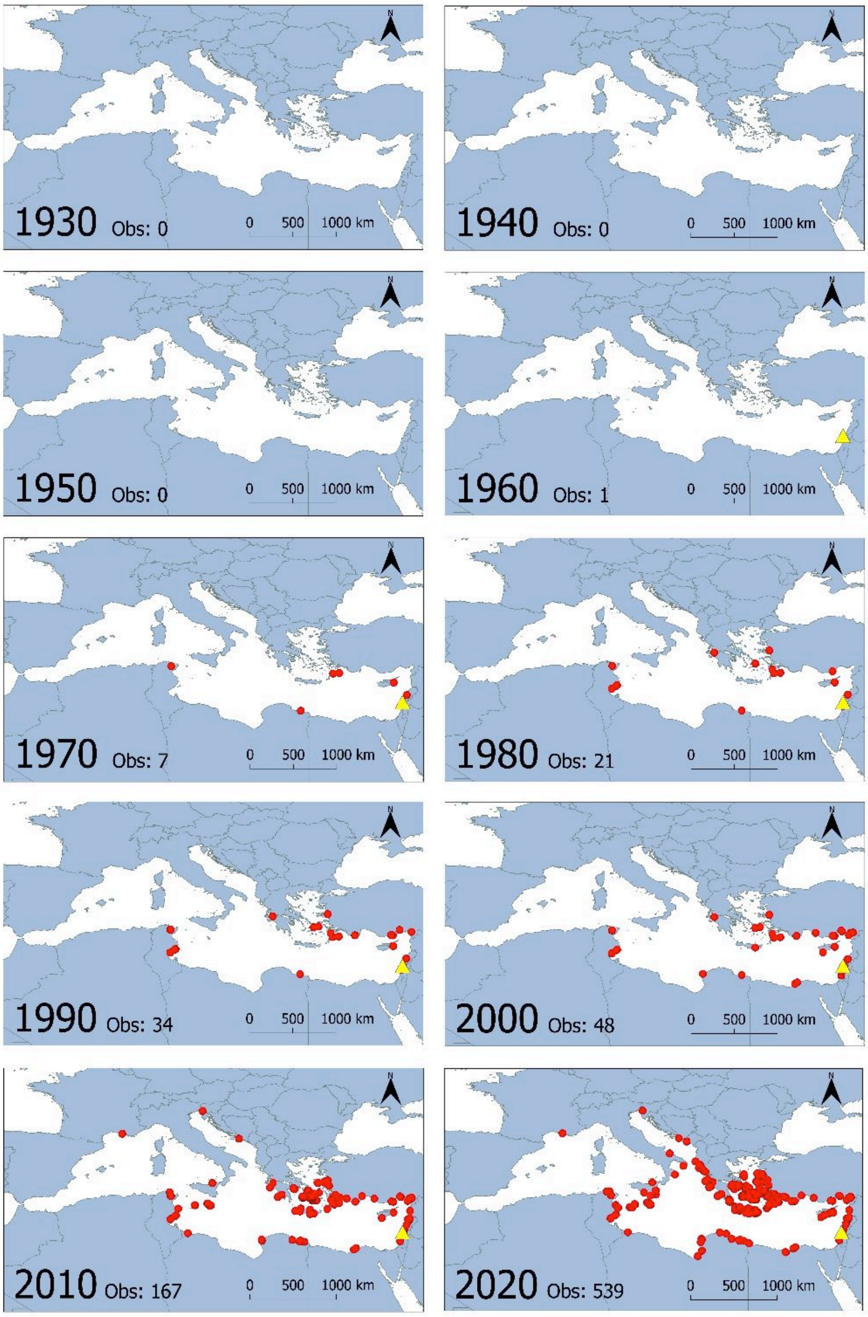


**Fig S3** Chronology of *Siganus luridus* invasion in the Mediterranean Sea. **First record** (yellow triangle): Israel, 1955. Cumulative occurrences are shown in 10-year intervals from 1930 to 2020. Data consisted of 539 georeferenced records pooled from bibliographic sources.

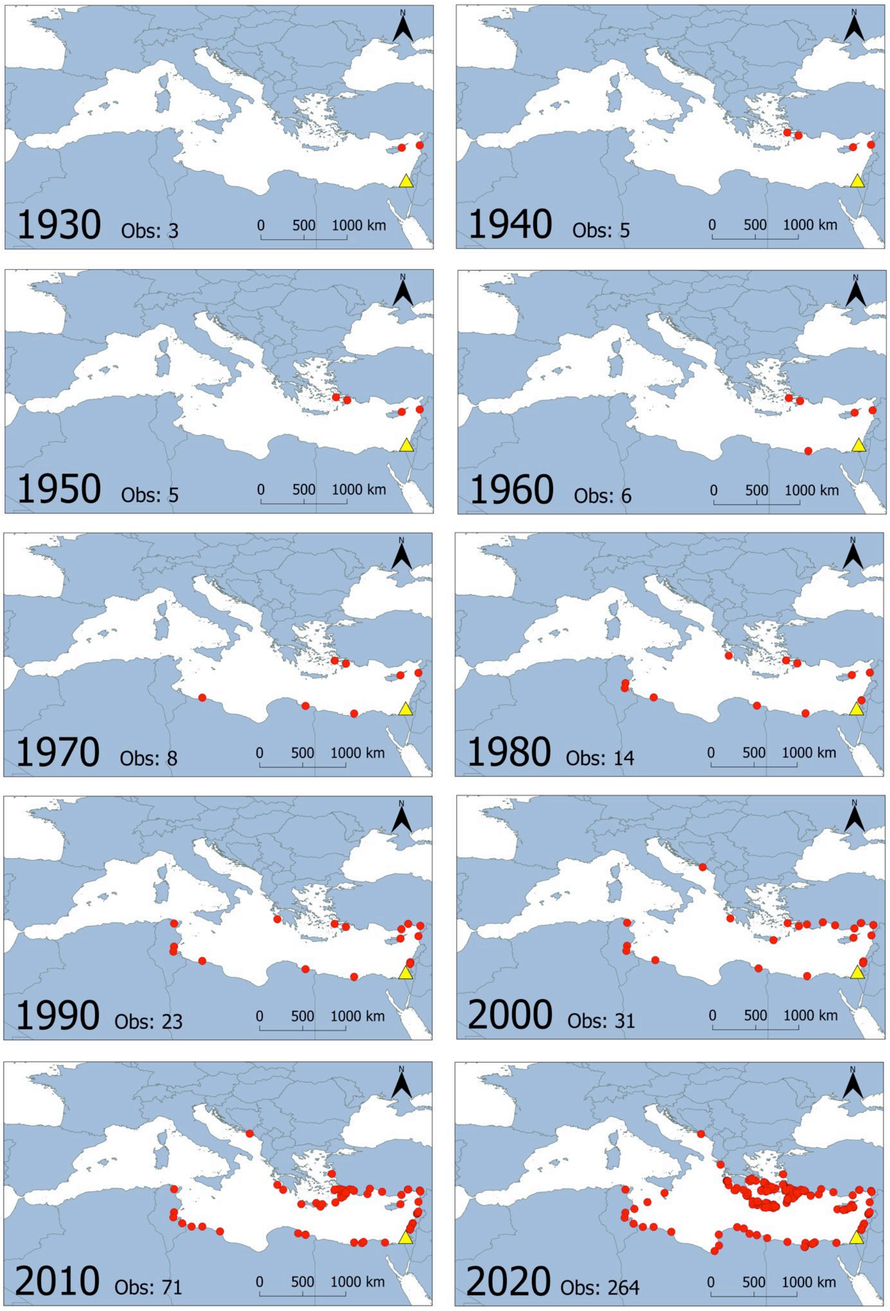


**Fig S4** Chronology of *Siganus rivulatus* invasion in the Mediterranean Sea. **First record** (yellow triangle): Israel, 1927. Cumulative occurrences are shown in 10-year intervals from 1930 to 2020. Data consisted of 264 georeferenced records pooled from bibliographic sources.

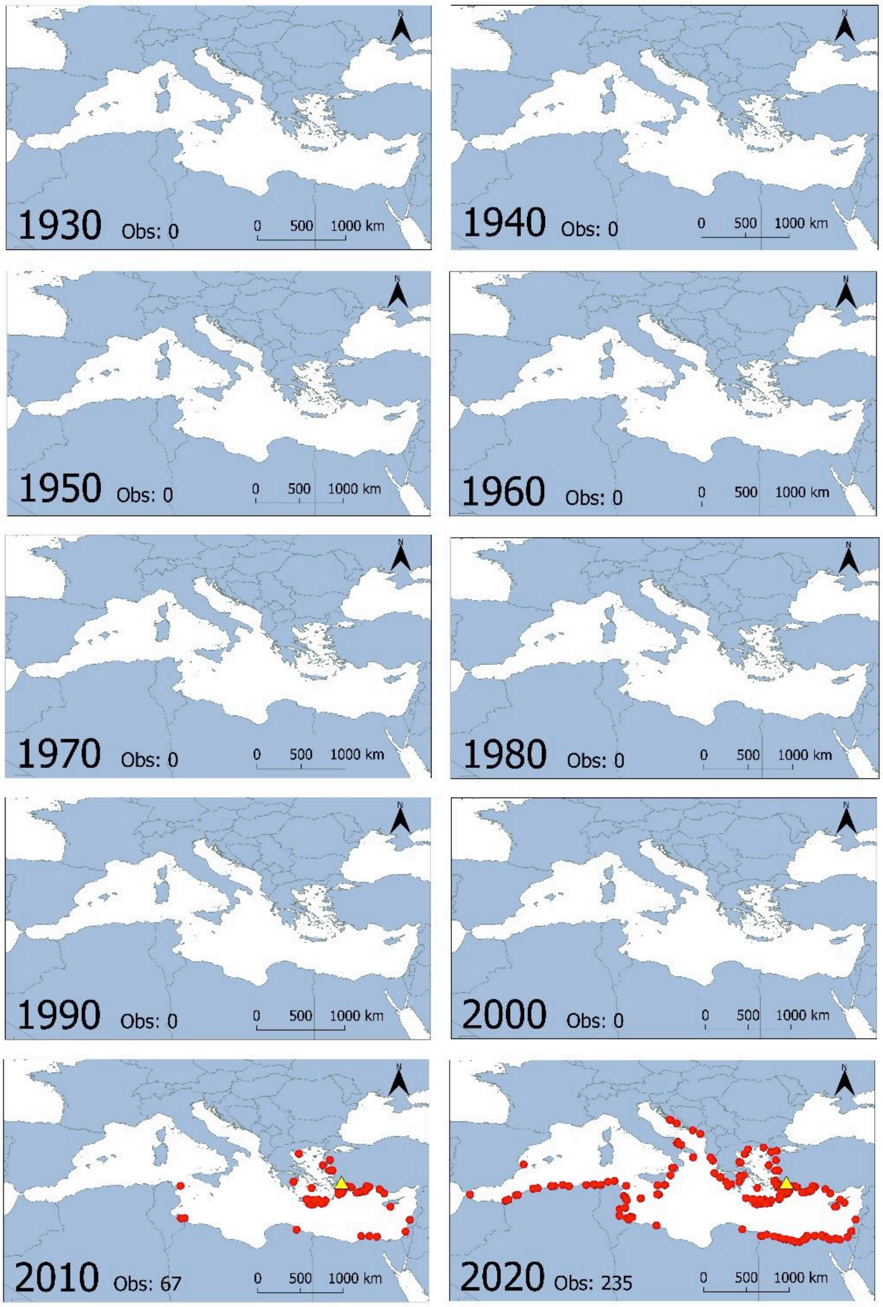


**Fig S5** Chronology of *Lagocephalus sceleratus* invasion in the Mediterranean Sea. **First record** (yellow triangle): Turkey, 2003. Cumulative occurrences are shown in 10-year intervals from 1930 to 2020. Data consisted of 235 georeferenced records pooled from bibliographic sources.

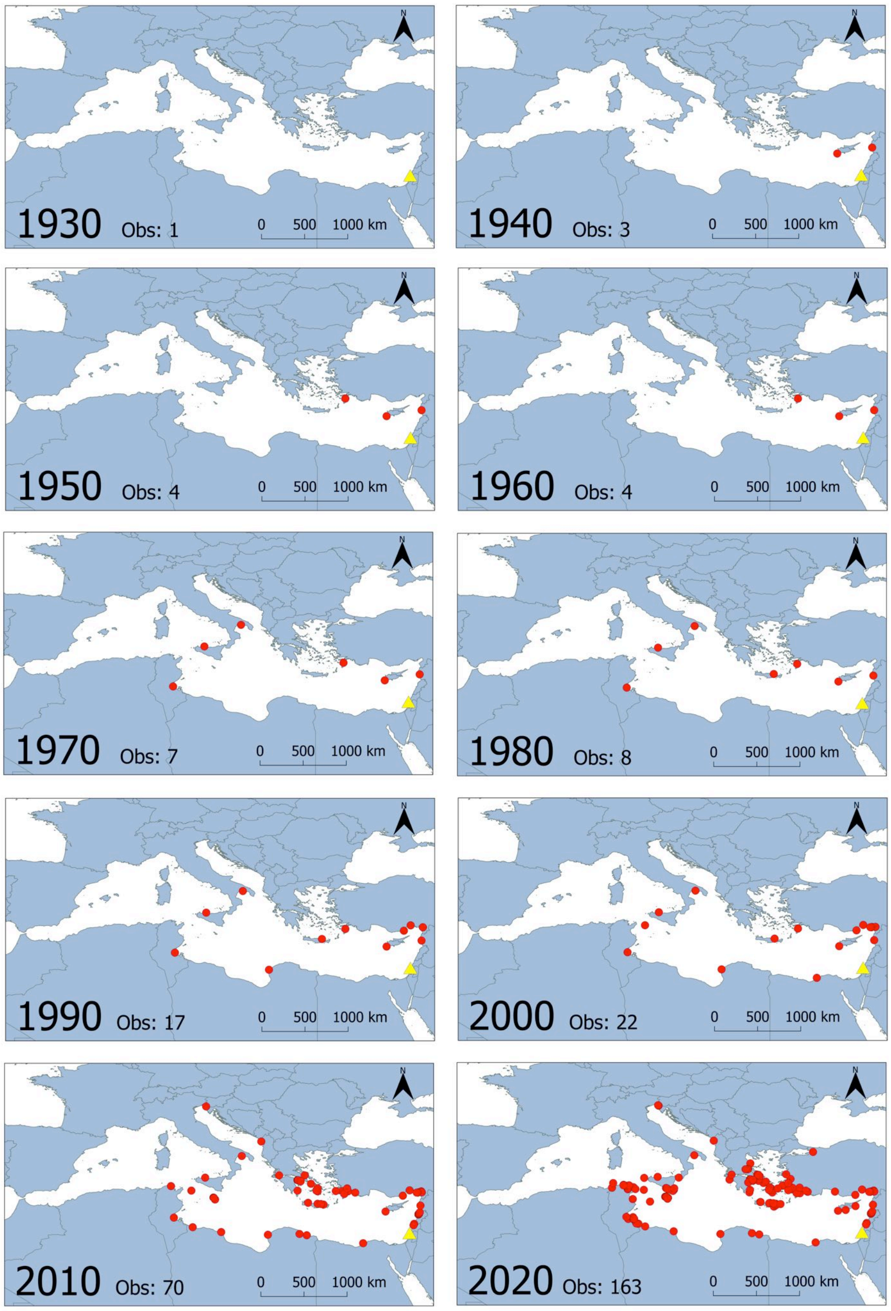


**Fig S6** Chronology of *Stephanolepis diaspros* invasion in the Mediterranean Sea. **First record** (yellow triangle): Israel, 1927. Cumulative occurrences are shown in 10-year intervals from 1930 to 2020. Data consisted of 163 georeferenced records pooled from bibliographic sources.

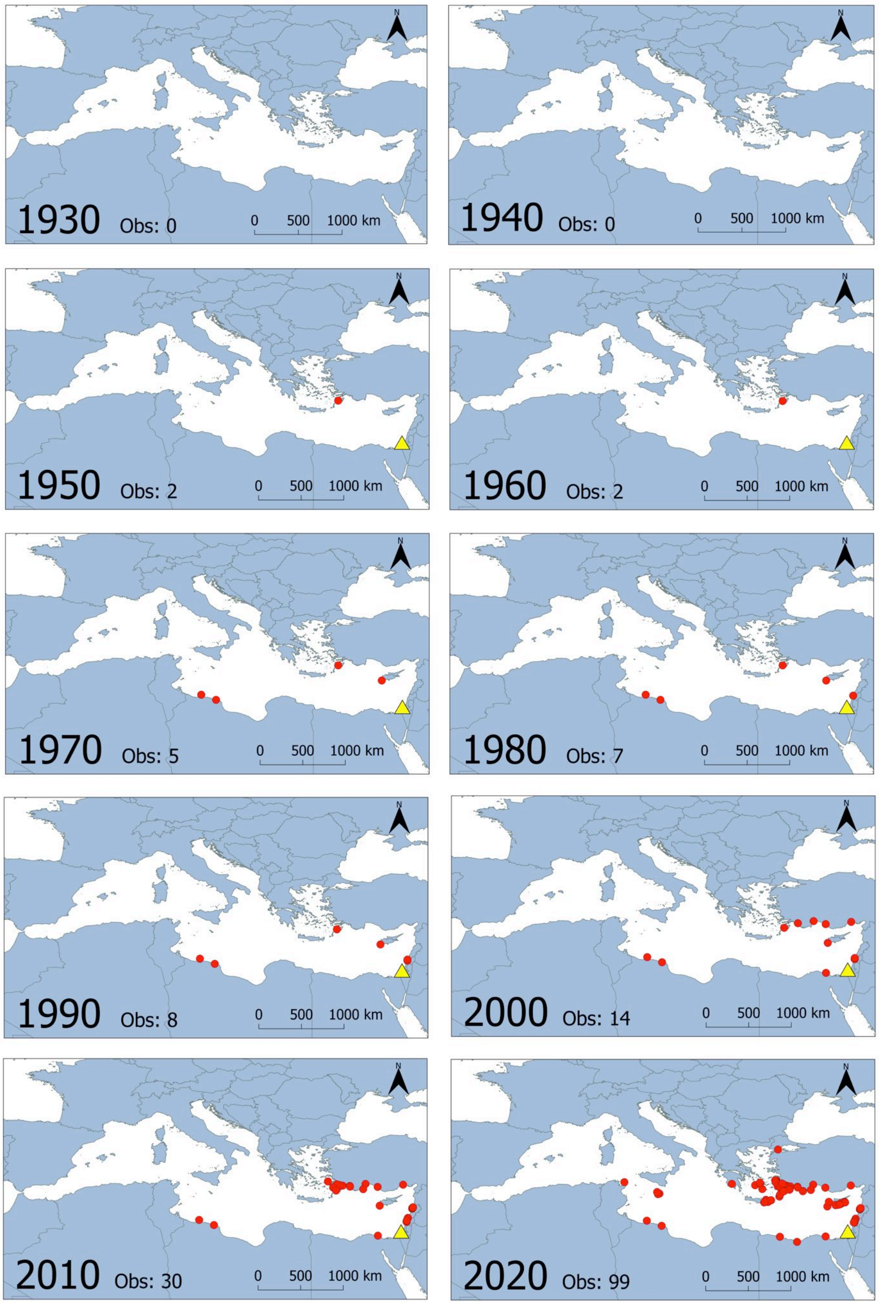


**Fig S7** Chronology of *Sargocentron rubrum* invasion in the Mediterranean Sea. **First record** (yellow triangle): Israel, 1945. Cumulative occurrences are shown in 10-year intervals from 1930 to 2020. Data consisted of 99 georeferenced records pooled from bibliographic sources.

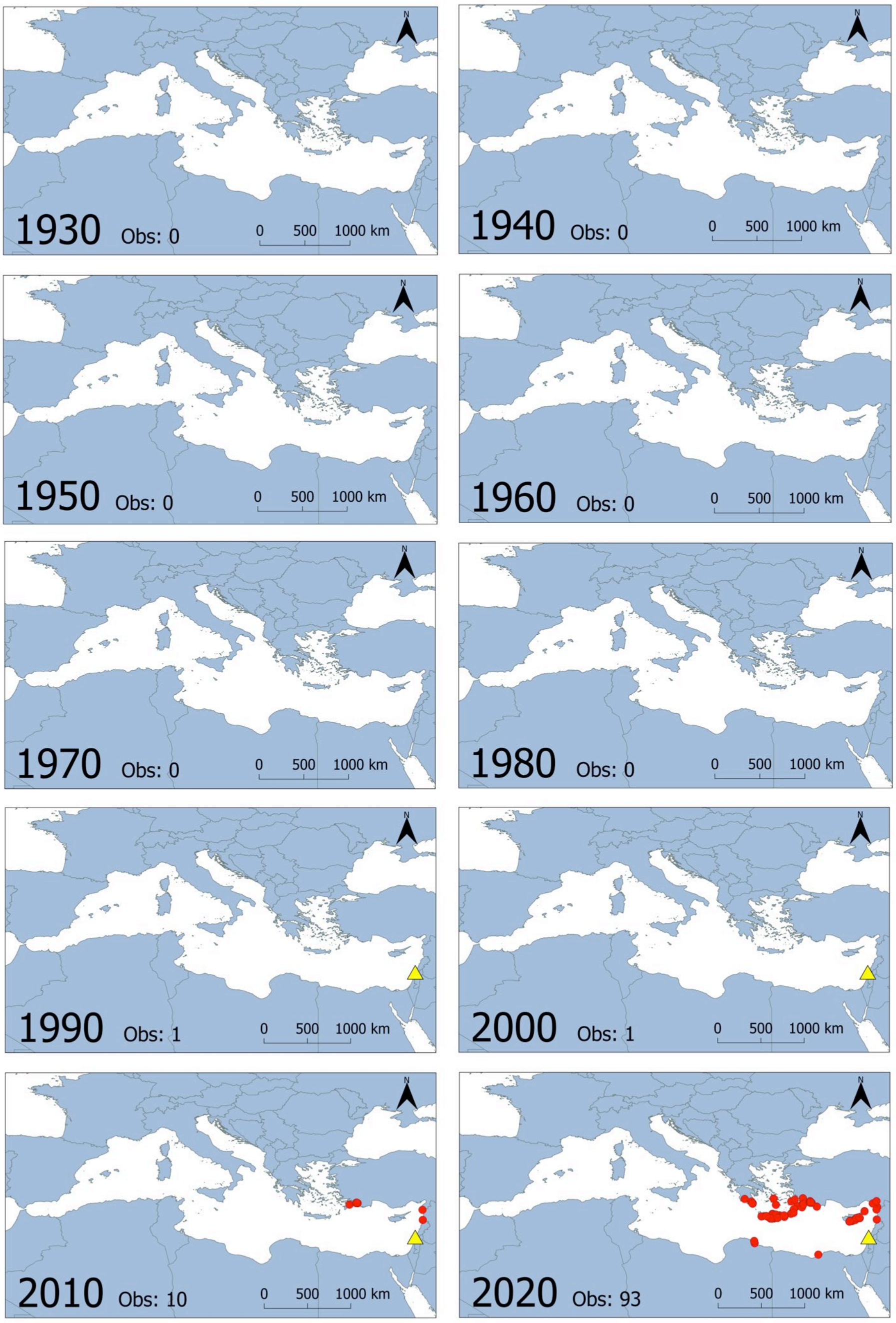


**Fig S8** Chronology of *Torquigener flavimaculosus* invasion in the Mediterranean Sea. **First record** (yellow triangle): Israel, 1987. Cumulative occurrences are shown in 10-year intervals from 1930 to 2020. Data consisted of 93 georeferenced records pooled from bibliographic sources.

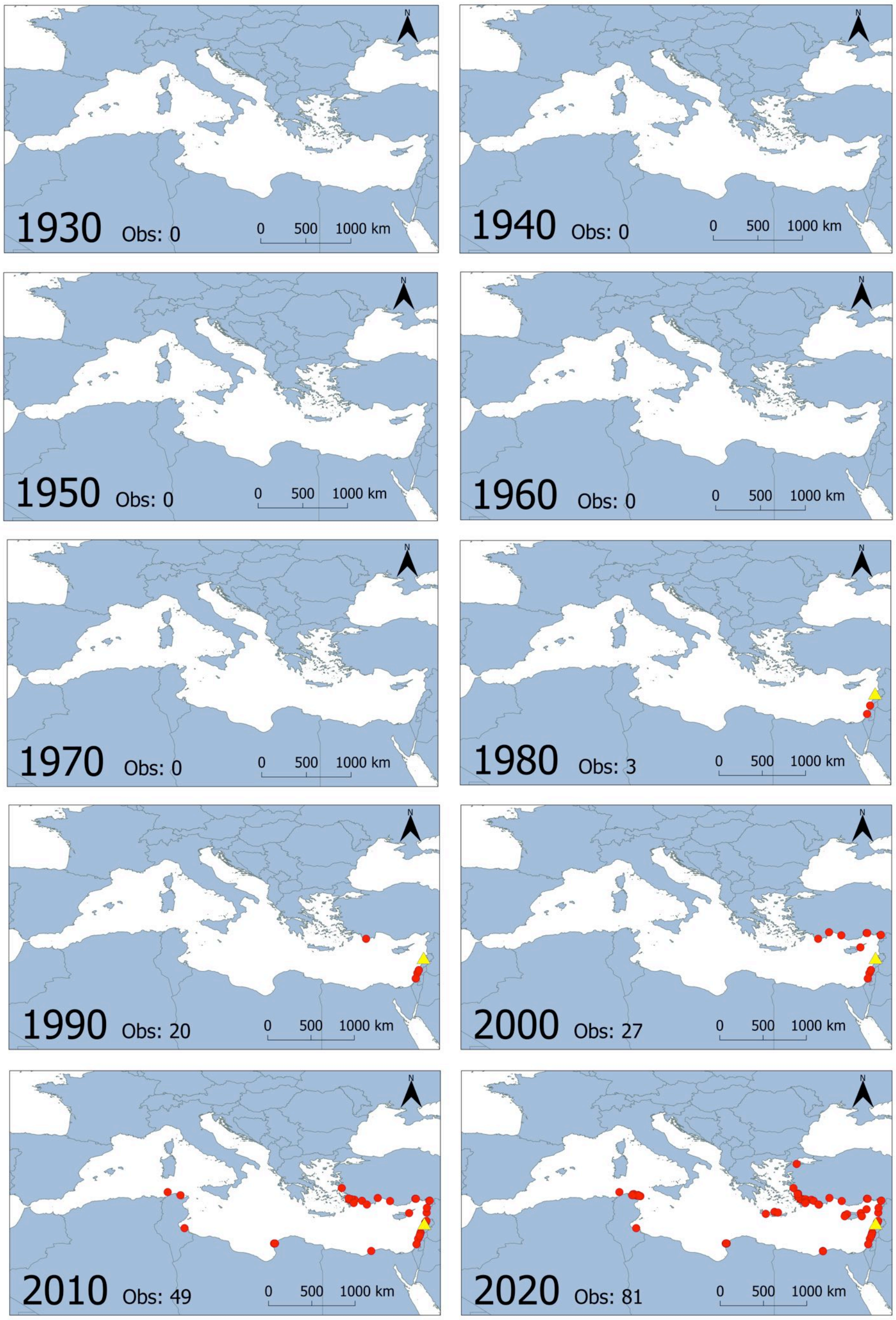


**Fig S9** Chronology of *Pempheris rhomboidea* invasion in the Mediterranean Sea. **First record** (yellow triangle): Lebanon, 1978. Cumulative occurrences are shown in 10-year intervals from 1930 to 2020. Data consisted of 81 georeferenced records pooled from bibliographic sources.

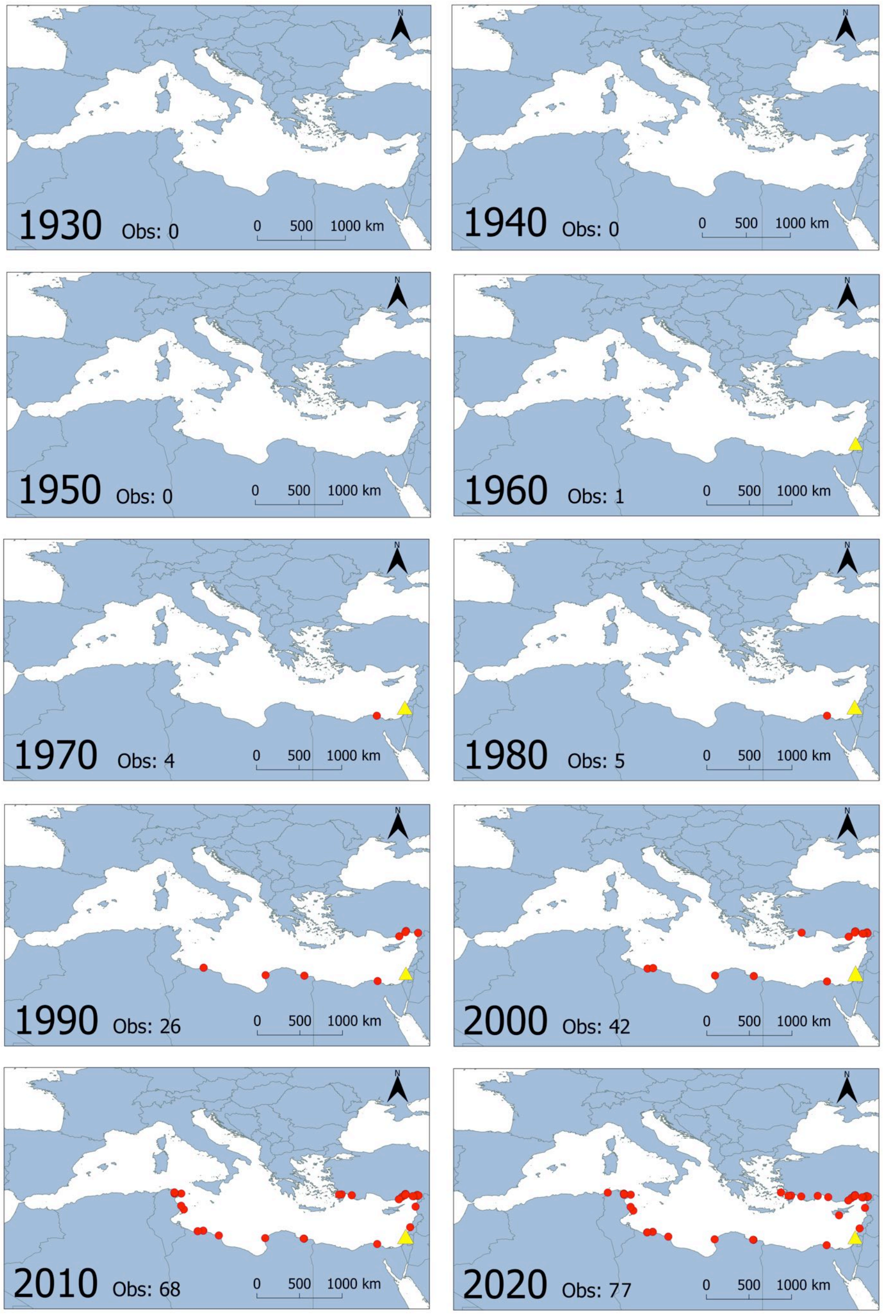


**Fig S10** Chronology of *Saurida lessepsianus* invasion in the Mediterranean Sea. **First record** (yellow triangle): Israel, 1952. Cumulative occurrences are shown in 10-year intervals from 1930 to 2020. Data consisted of 77 georeferenced records pooled from bibliographic sources.

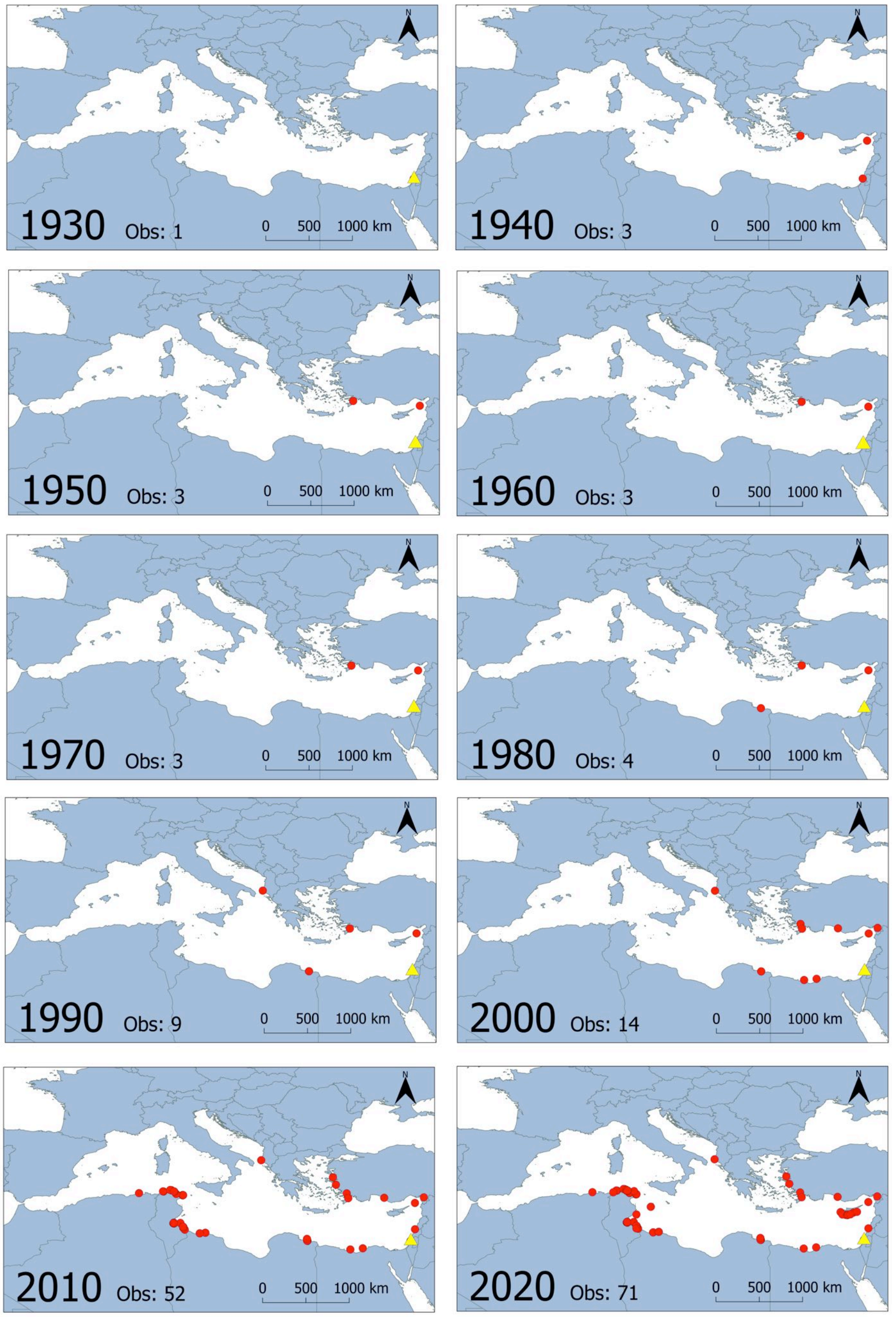


**Fig S11** Chronology of *Hemiramphus far* invasion in the Mediterranean Sea. **First record** (yellow triangle): Israel, 1927. Cumulative occurrences are shown in 10-year intervals from 1930 to 2020. Data consisted of 71 georeferenced records pooled from bibliographic sources.

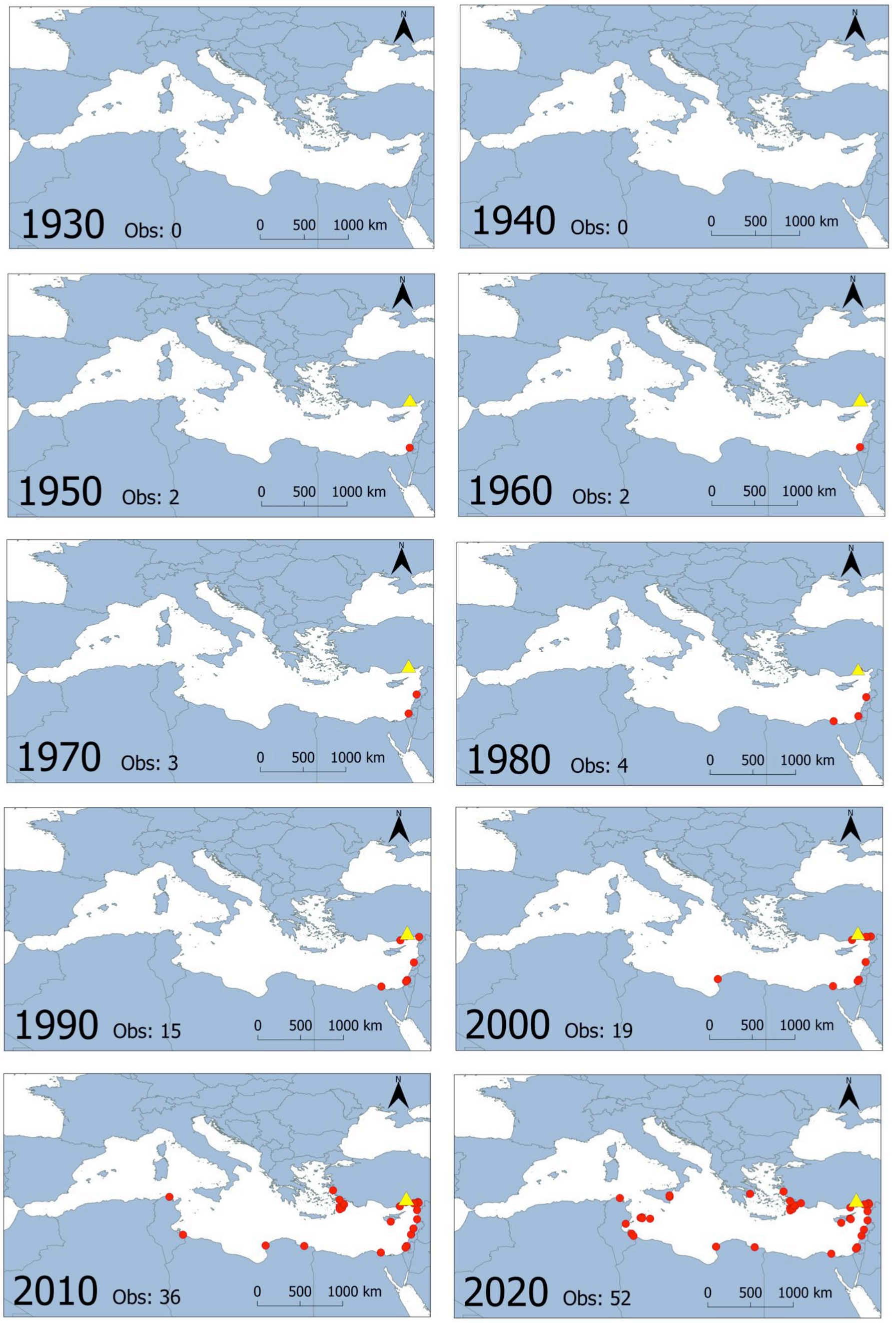


**Fig S12** Chronology of *Upeneus pori* invasion in the Mediterranean Sea. **First record** (yellow triangle): Turkey, 1942. Cumulative occurrences are shown in 10-year intervals from 1930 to 2020. Data consisted of 52 georeferenced records pooled from bibliographic sources.

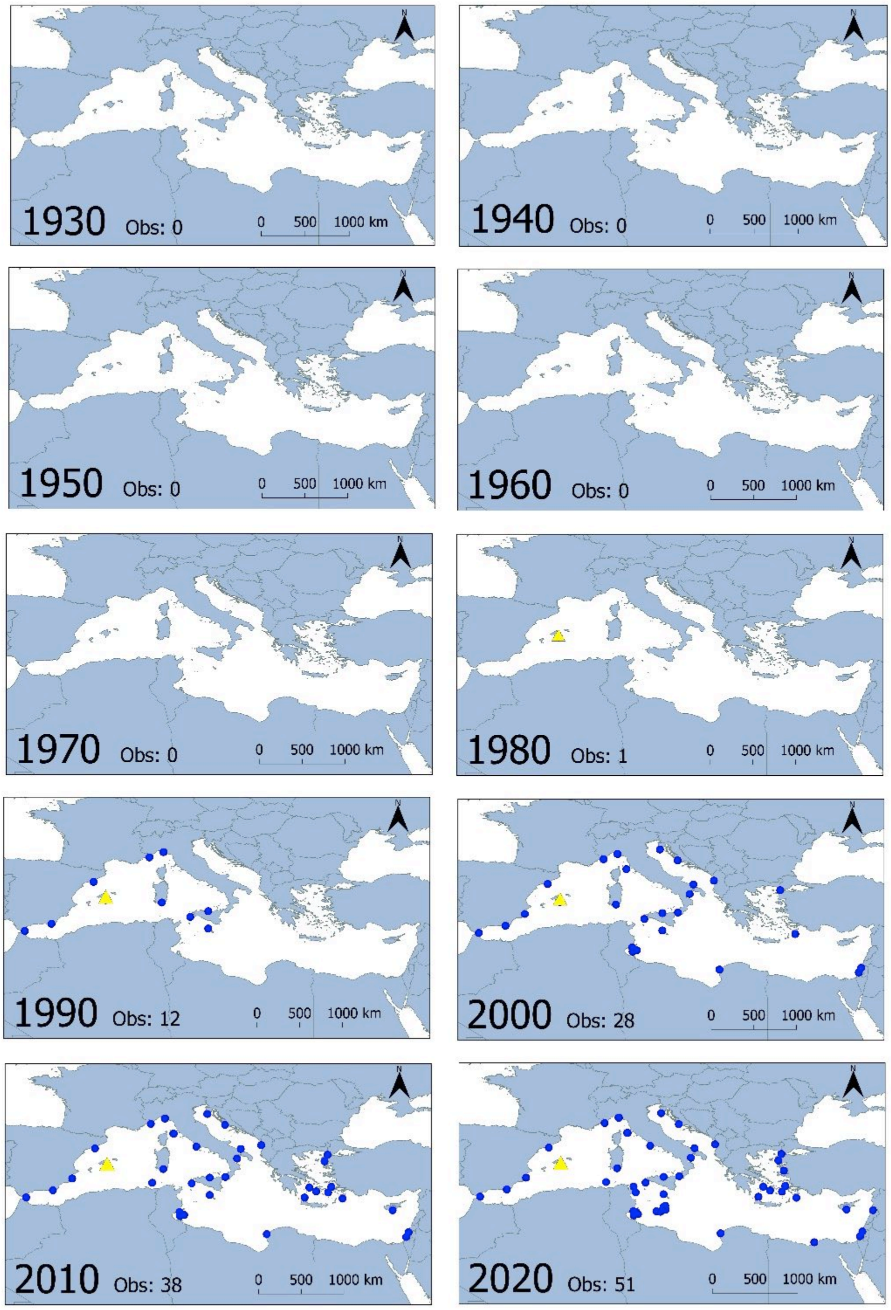


**Fig S13** Chronology of *Sphoeroides pachygaster* invasion in the Mediterranean Sea. **First record** (yellow triangle): Spain, 1979. Cumulative occurrences are shown in 10-year intervals from 1930 to 2020. Data consisted of 51 georeferenced records pooled from bibliographic sources.

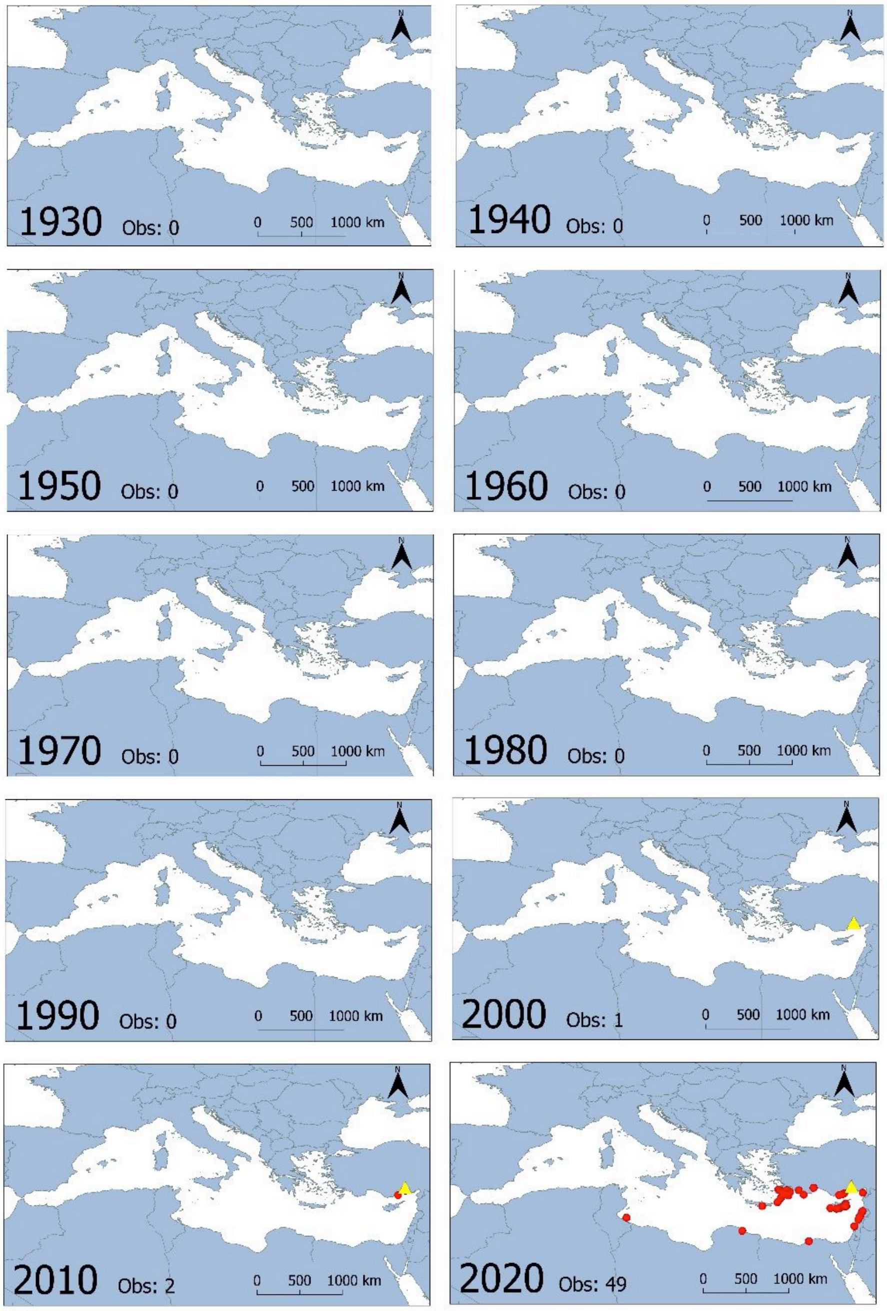


**Fig S14** Chronology of *Parupeneus forsskali* invasion in the Mediterranean Sea. **First record** (yellow triangle): Turkey, 2000. Cumulative occurrences are shown in 10-year intervals from 1930 to 2020. Data consisted of 49 georeferenced records pooled from bibliographic sources.

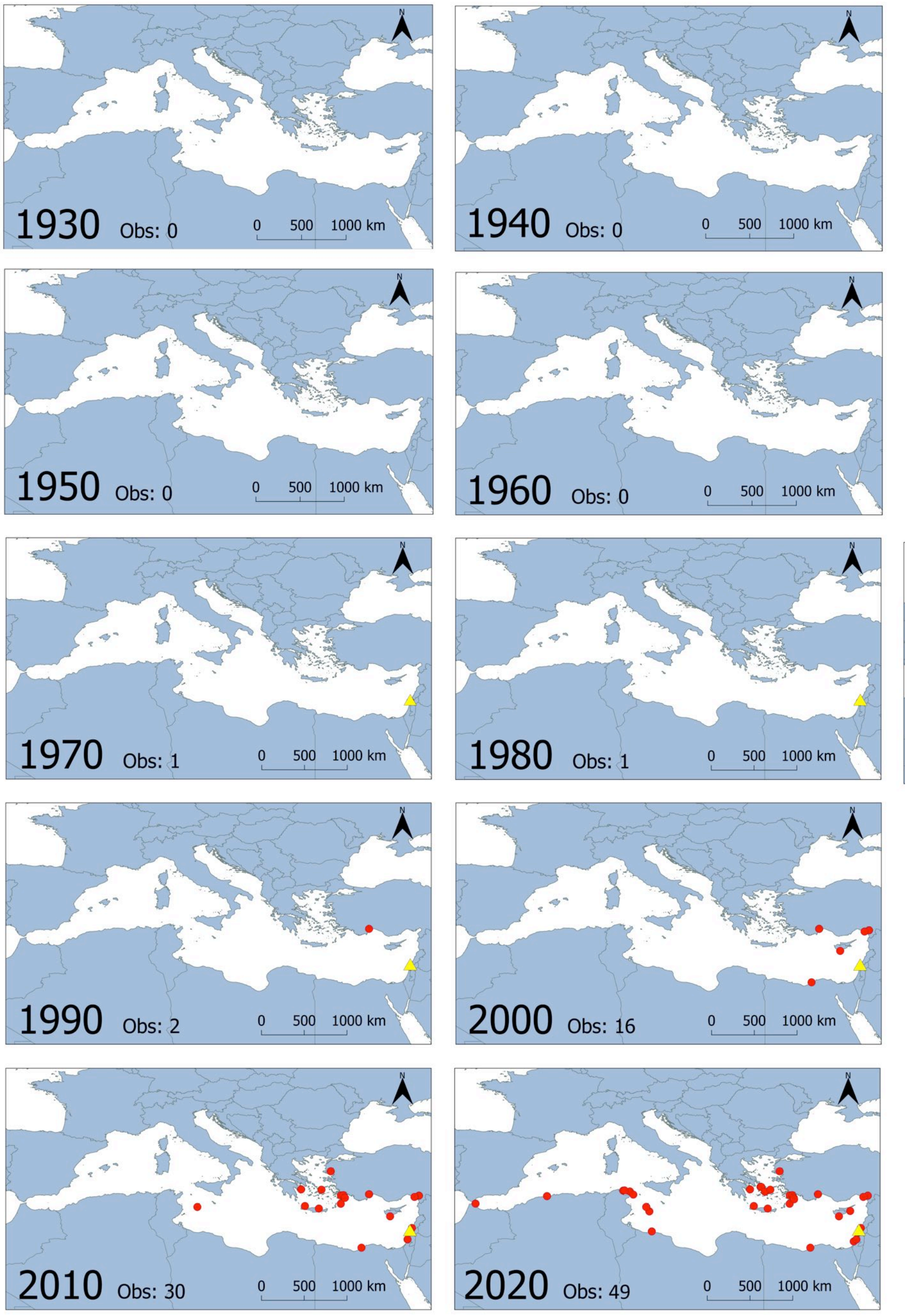


**Fig S15** Chronology of *Etrumeus golanii* invasion in the Mediterranean Sea. **First record** (yellow triangle): Israel, 1961. Cumulative occurrences are shown in 10-year intervals from 1930 to 2020. Data consisted of 49 georeferenced records pooled from bibliographic sources.

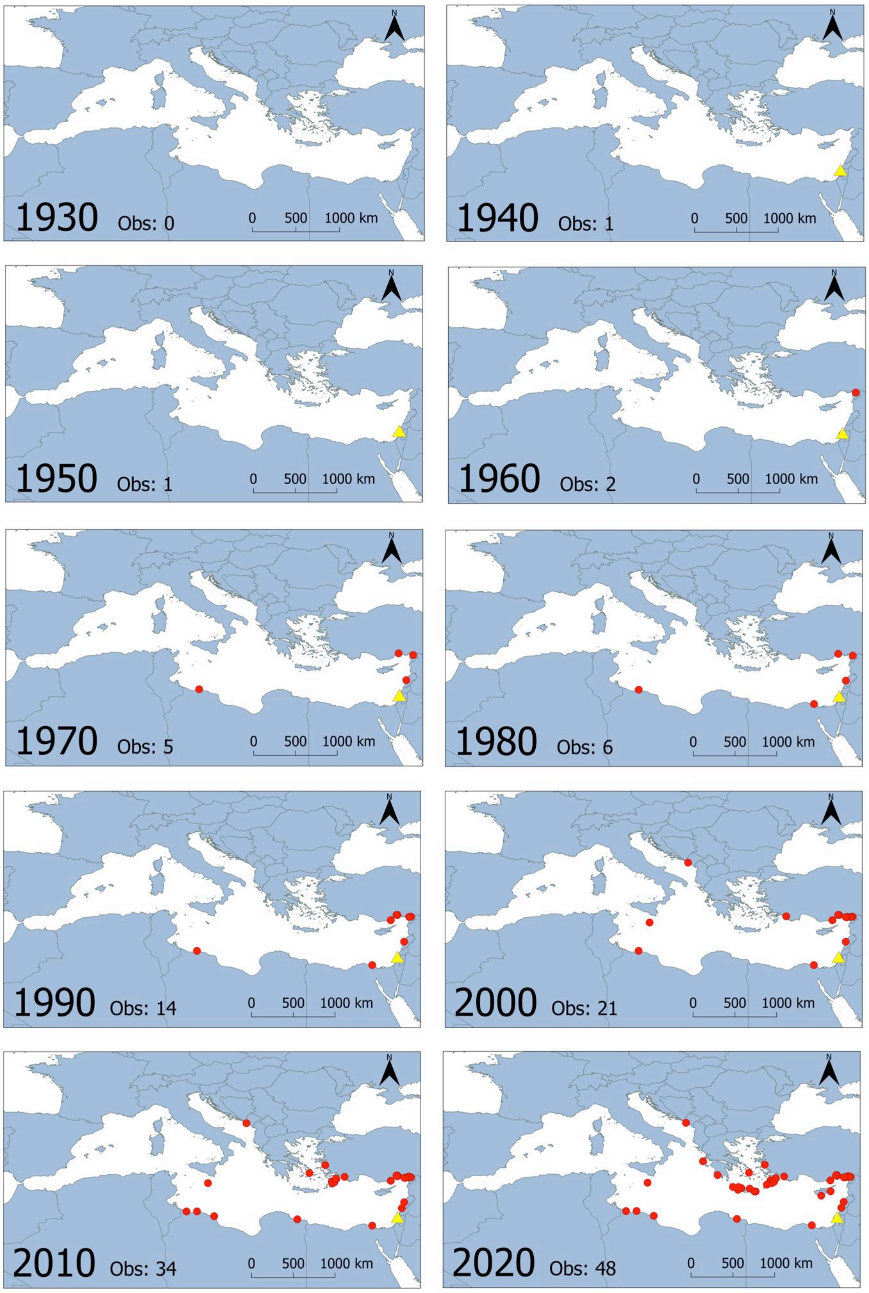


**Fig S16** Chronology of *Sphyraena chrysotaenia* invasion in the Mediterranean Sea. **First record** (yellow triangle): Israel, 1931. Cumulative occurrences are shown in 10-year intervals from 1930 to 2020. Data consisted of 48 georeferenced records pooled from bibliographic sources.

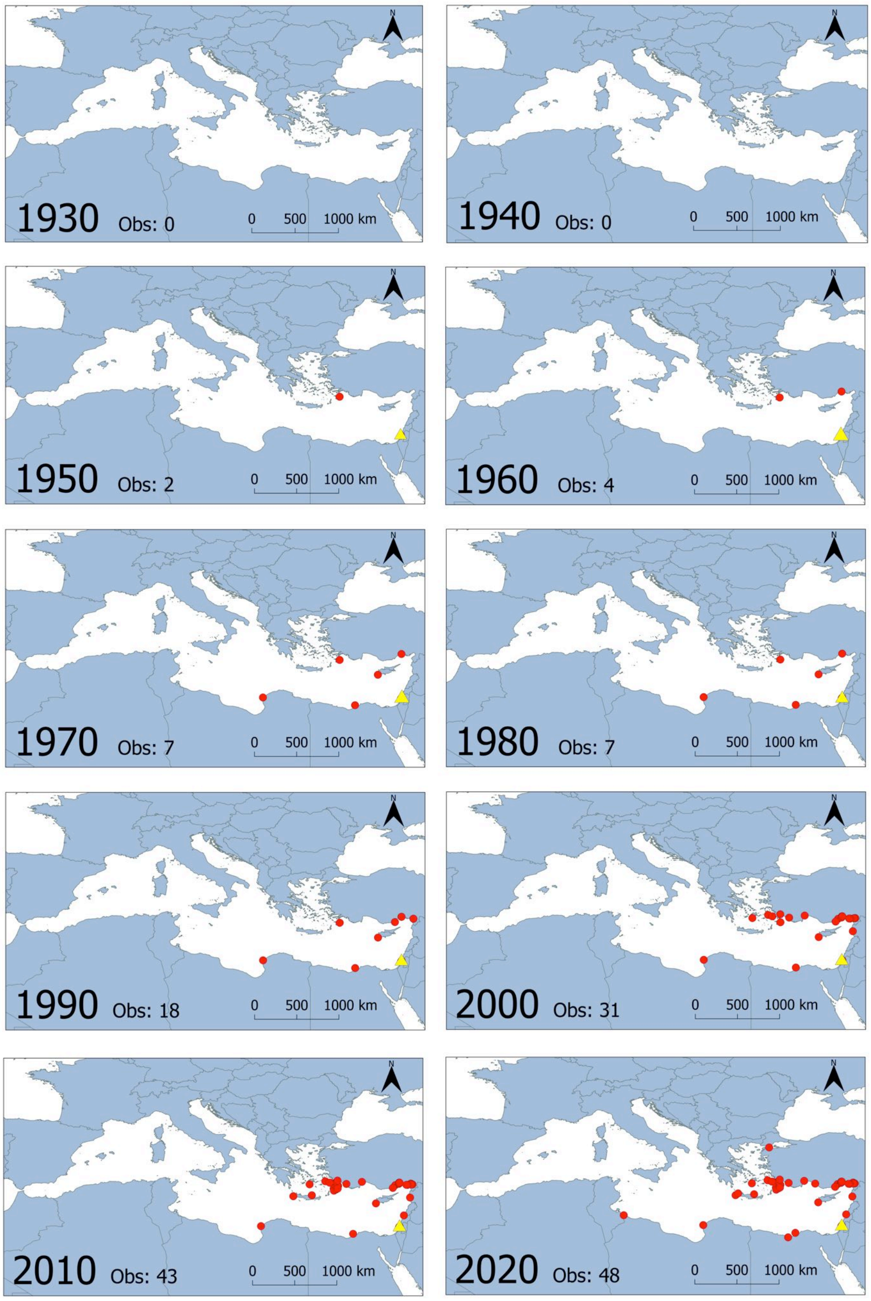


**Fig S17** Chronology of *Upeneus moluccensis* invasion in the Mediterranean Sea. **First record** (yellow triangle): Israel, 1946. Cumulative occurrences are shown in 10-year intervals from 1930 to 2020. Data consisted of 48 georeferenced records pooled from bibliographic sources.

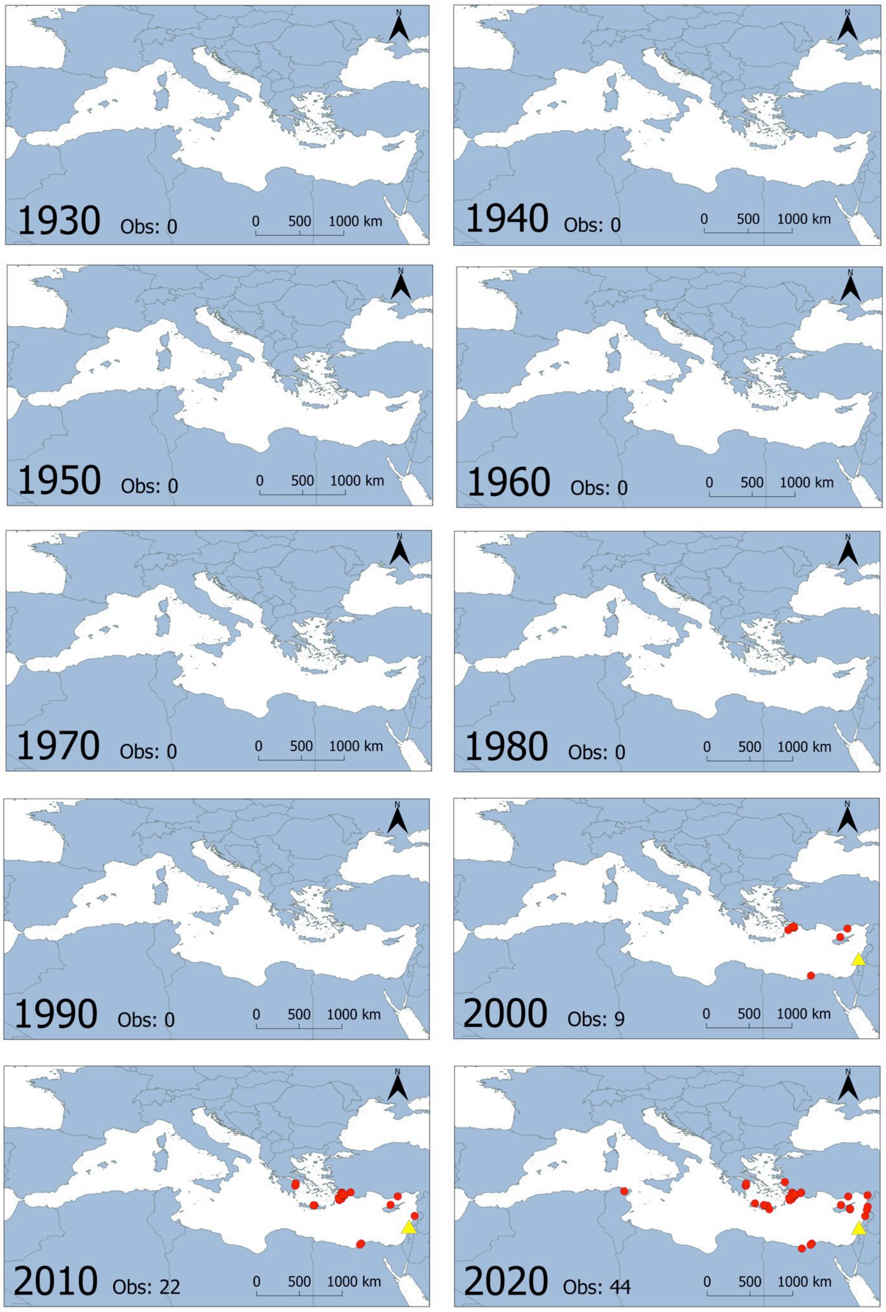


**Fig S18** Chronology of *Pteragogus trispilus* invasion in the Mediterranean Sea. **First record** (yellow triangle): Israel, 1991. Cumulative occurrences are shown in 10-year intervals from 1930 to 2020. Data consisted of 44 georeferenced records pooled from bibliographic sources.

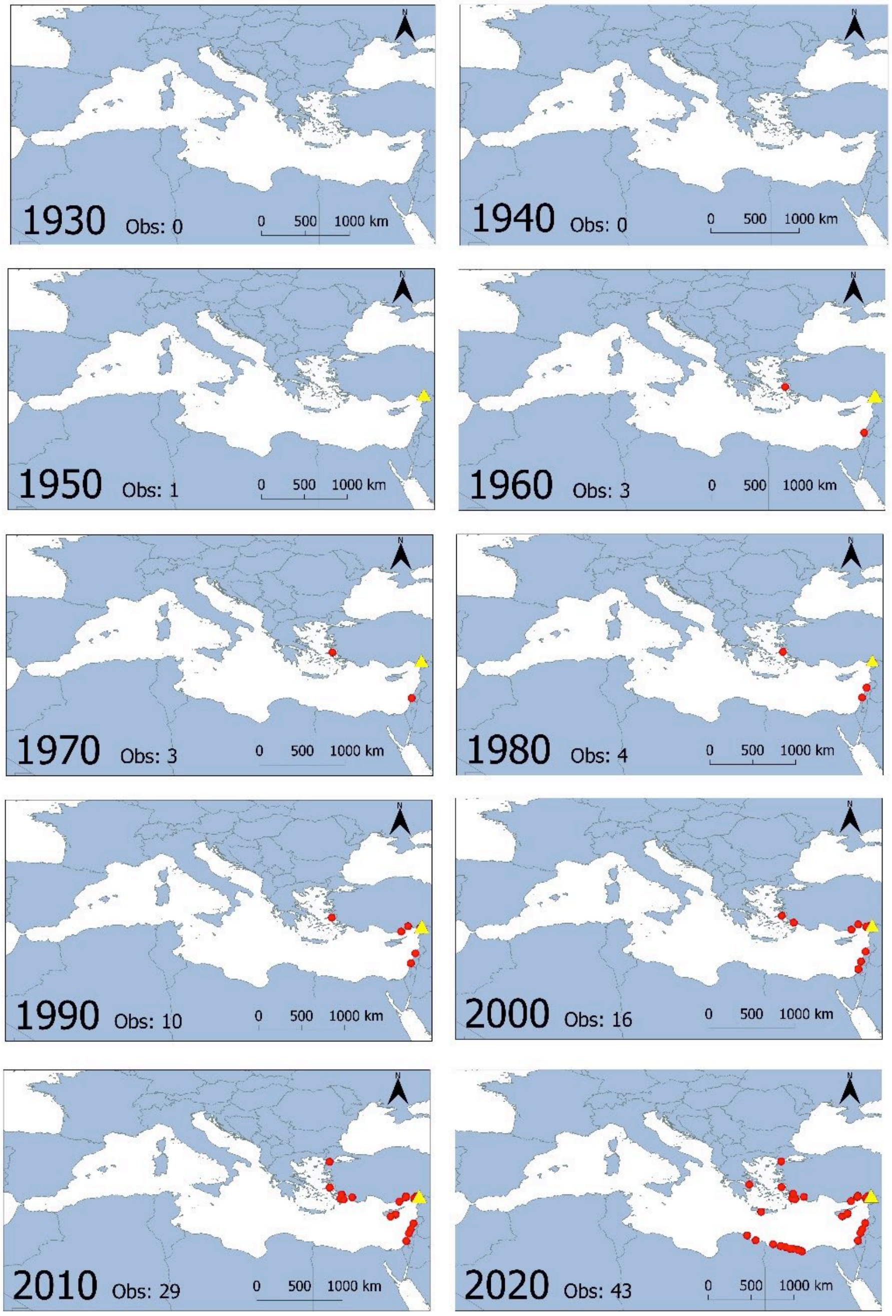


**Fig S19** Chronology of *Lagocephalus guentheri* invasion in the Mediterranean Sea. **First record** (yellow triangle): Turkey, 1950. Cumulative occurrences are shown in 10-year intervals from 1930 to 2020. Data consisted of 43 georeferenced records pooled from bibliographic sources.

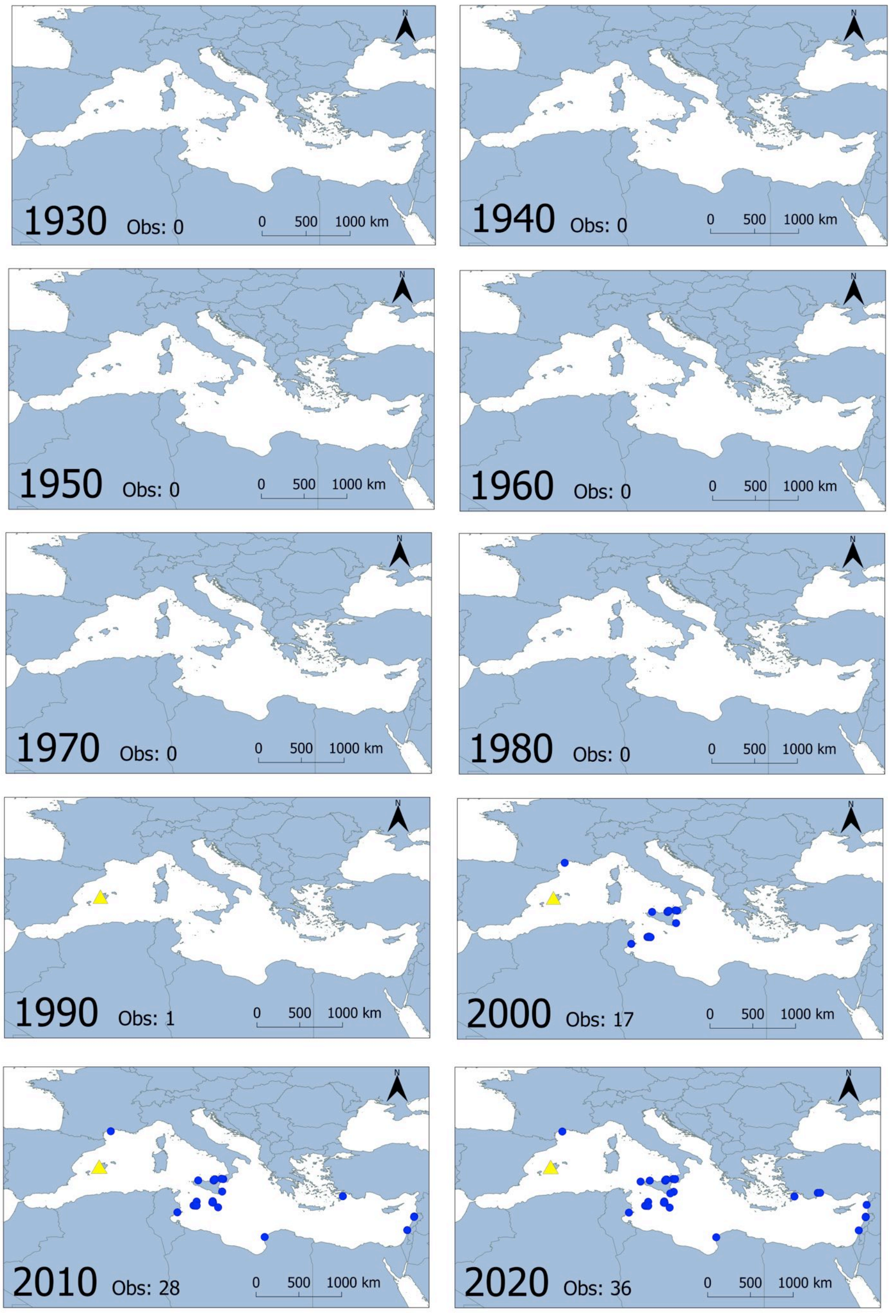


**Fig S20** Chronology of *Seriola fasciata* invasion in the Mediterranean Sea. **First record** (yellow triangle): Spain, 1989. Cumulative occurrences are shown in 10-year intervals from 1930 to 2020. Data consisted of 36 georeferenced records pooled from bibliographic sources.

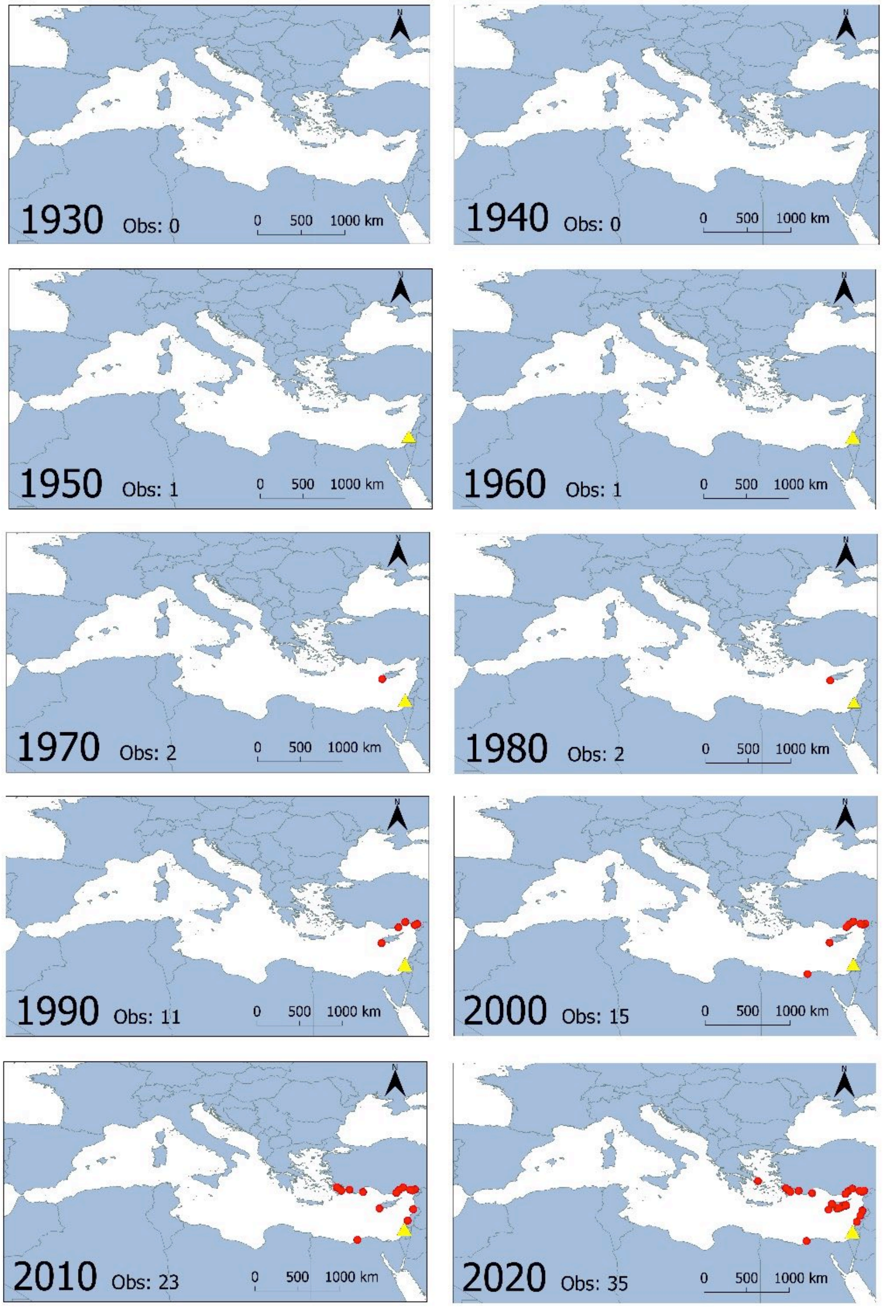


**Fig S21** Chronology of *Apogonichthyoides pharaonis* invasion in the Mediterranean Sea. **First record** (yellow triangle): Israel, 1947. Cumulative occurrences are shown in 10-year intervals from 1930 to 2020. Data consisted of 35 georeferenced records pooled from bibliographic sources.

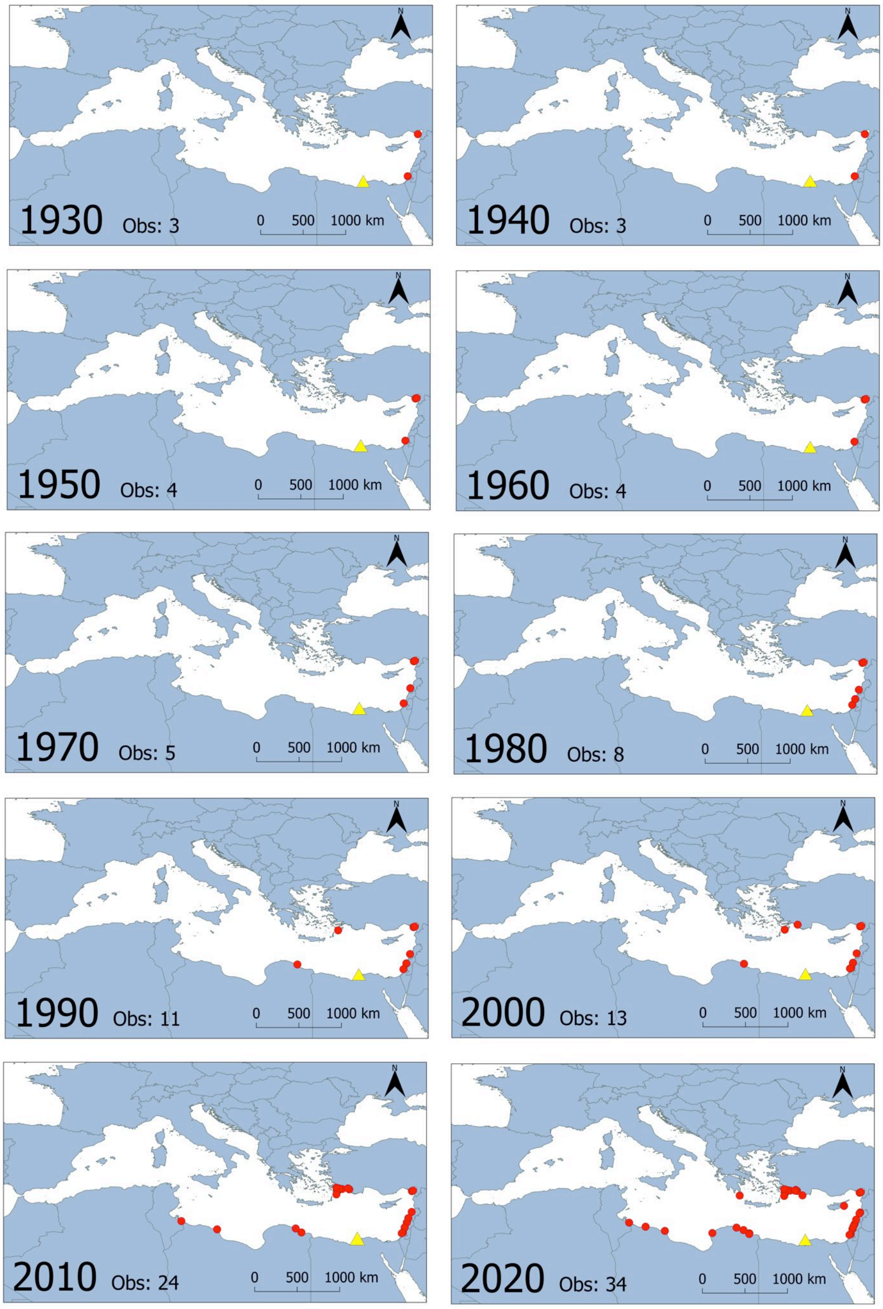


**Fig S22** Chronology of *Atherinomorus forksalii* invasion in the Mediterranean Sea. **First record** (yellow triangle): Egypt, 1902. Cumulative occurrences are shown in 10-year intervals from 1930 to 2020. Data consisted of 34 georeferenced records pooled from bibliographic sources.

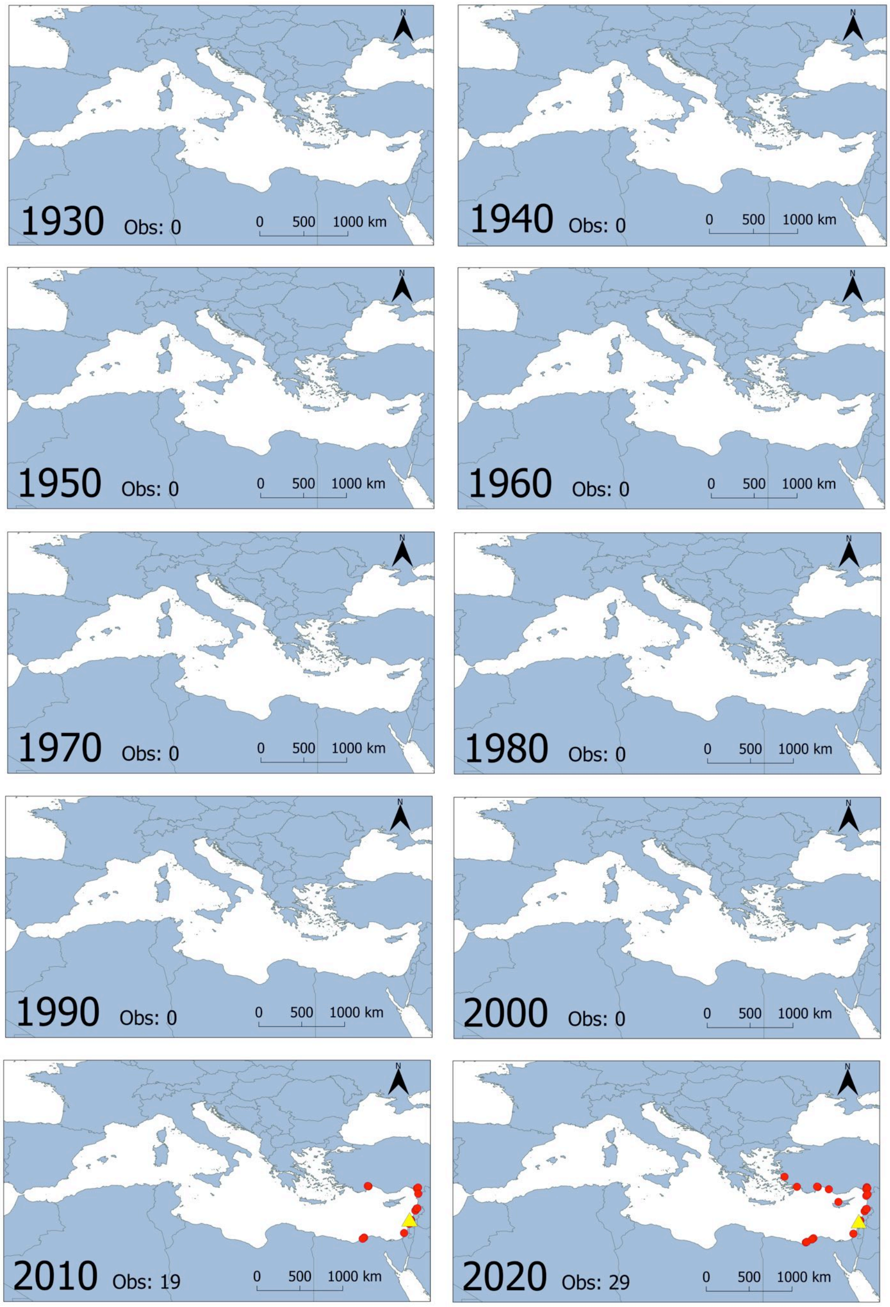


**Fig S23** Chronology of *Nemipterus randalli* invasion in the Mediterranean Sea. **First record** (yellow triangle): Israel, 2005. Cumulative occurrences are shown in 10-year intervals from 1930 to 2020. Data consisted of 29 georeferenced records pooled from bibliographic sources.

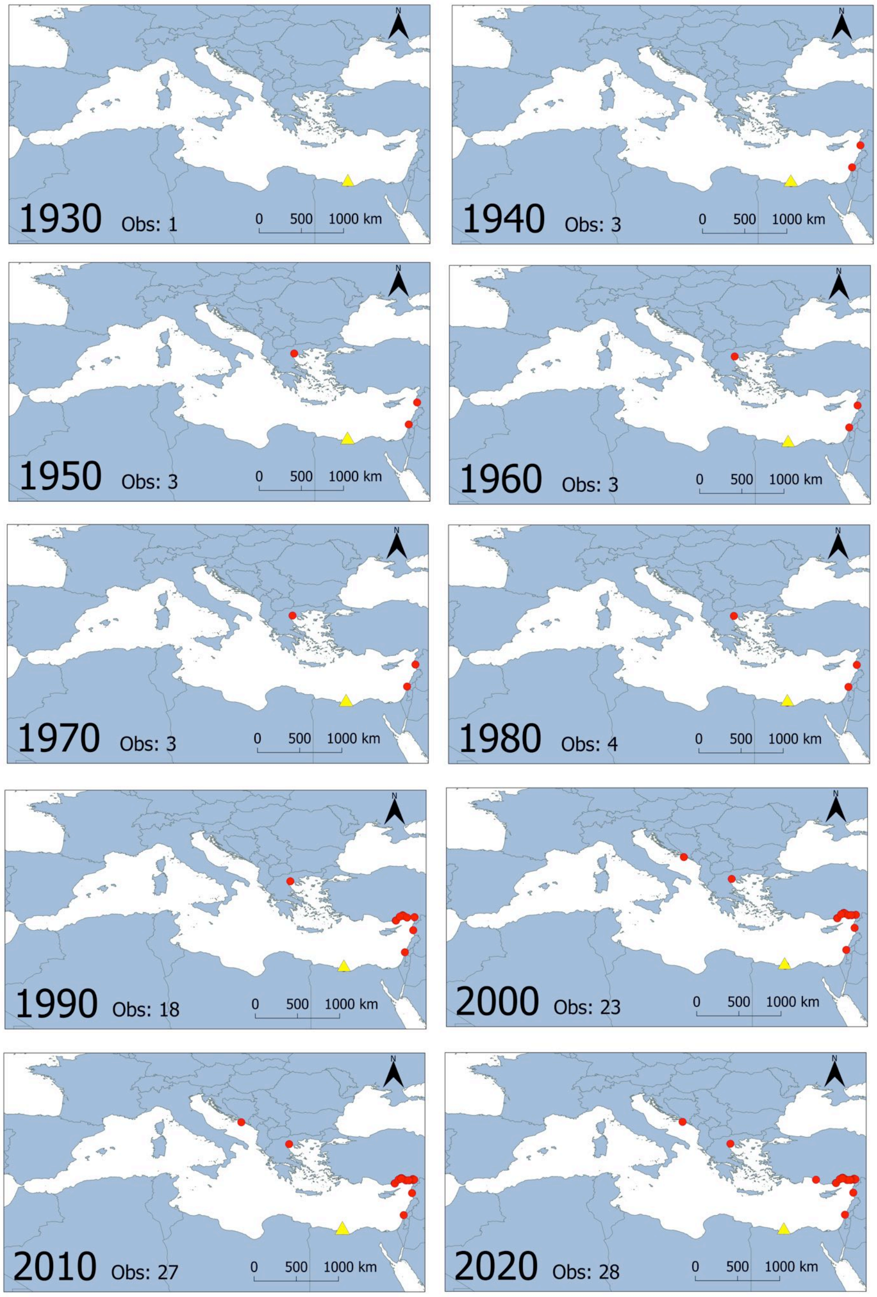


**Fig S24** Chronology of *Equulites klunzingeri* invasion in the Mediterranean Sea. **First record** (yellow triangle): Egypt, 1924. Cumulative occurrences are shown in 10-year intervals from 1930 to 2020. Data consisted of 28 georeferenced records pooled from bibliographic sources.

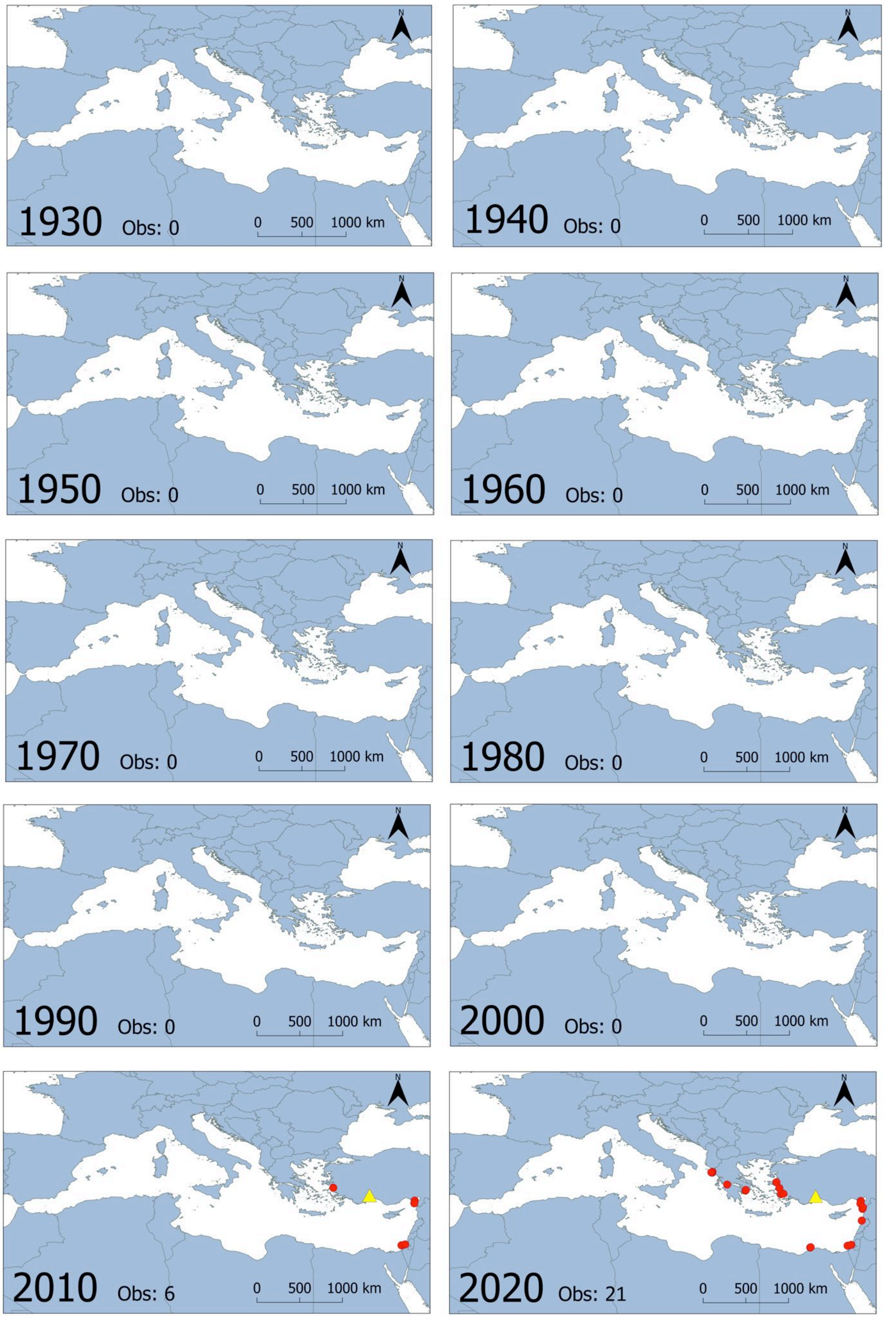


**Fig S25** Chronology of *Bregmaceros nectabanus* invasion in the Mediterranean Sea. **First record** (yellow triangle): Turkey, 2002. Cumulative occurrences are shown in 10-year intervals from 1930 to 2020. Data consisted of 21 georeferenced records pooled from bibliographic sources.

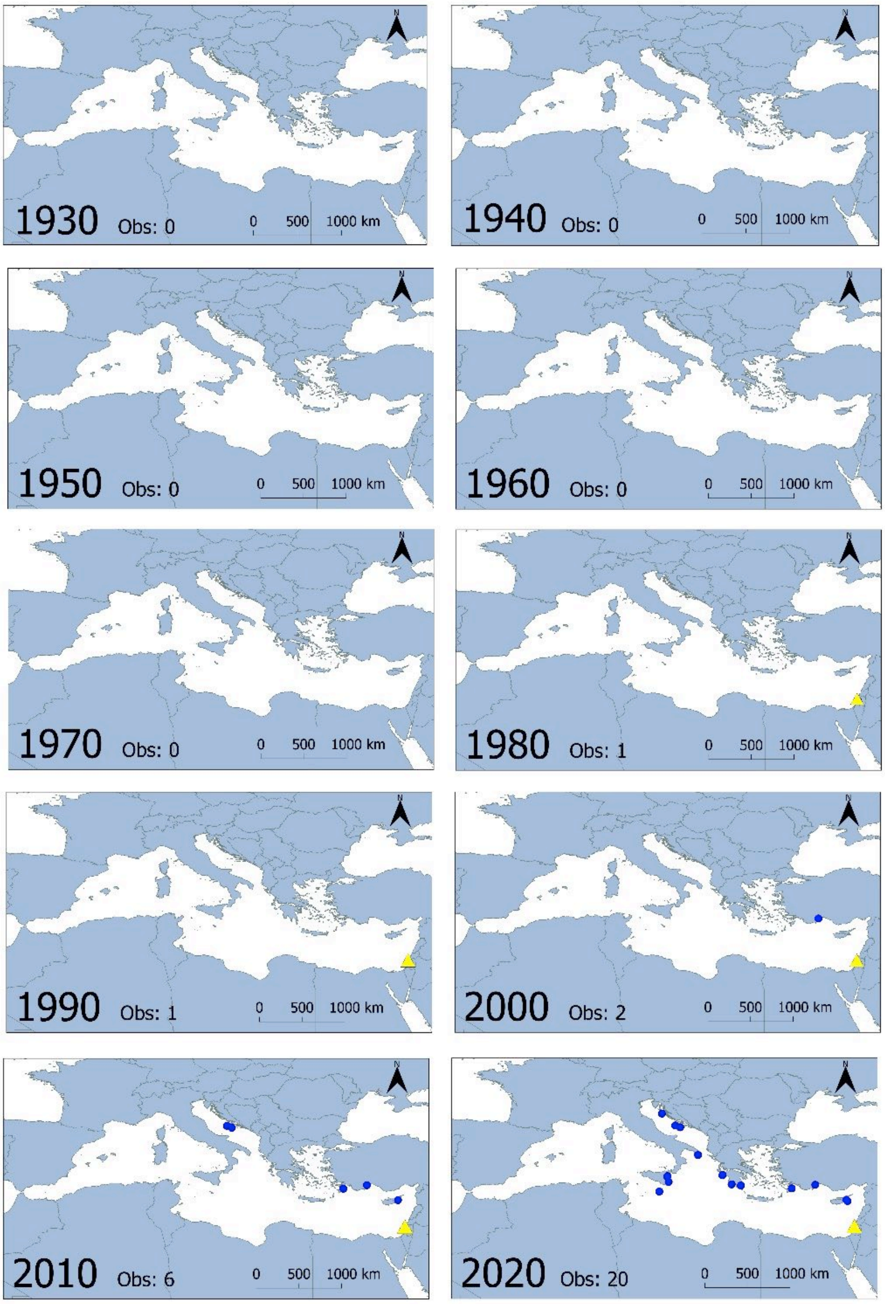


**Fig S26** Chronology of *Enchelycore anatina* invasion in the Mediterranean Sea. **First record** (yellow triangle): Israel, 1979. Cumulative occurrences are shown in 10-year intervals from 1930 to 2020. Data consisted of 20 georeferenced records pooled from bibliographic sources.
