# Supplementary material for: Spatio‐temporal dynamics of exotic fish species in the Mediterranean Sea: Over a century of invasion reconstructed
Source: Glob Chang Biol. 2022 Sep 2;28(21):6268–79. doi: 10.1111/gcb.16362 (PMC9826093; doi:10.1111/gcb.16362)

**Appendix 2.**

Violin plots showing how data records for each species (CAN in Red, HM in yellow and NRE in Blue) vary along longitude, using density curves based on kernel probability. The boxplot summarizes the centre and spread of the distribution: centre of the boxes represents the median and the length of the box represents the interquartile range. Records outside the interquartile are represented by black dots.


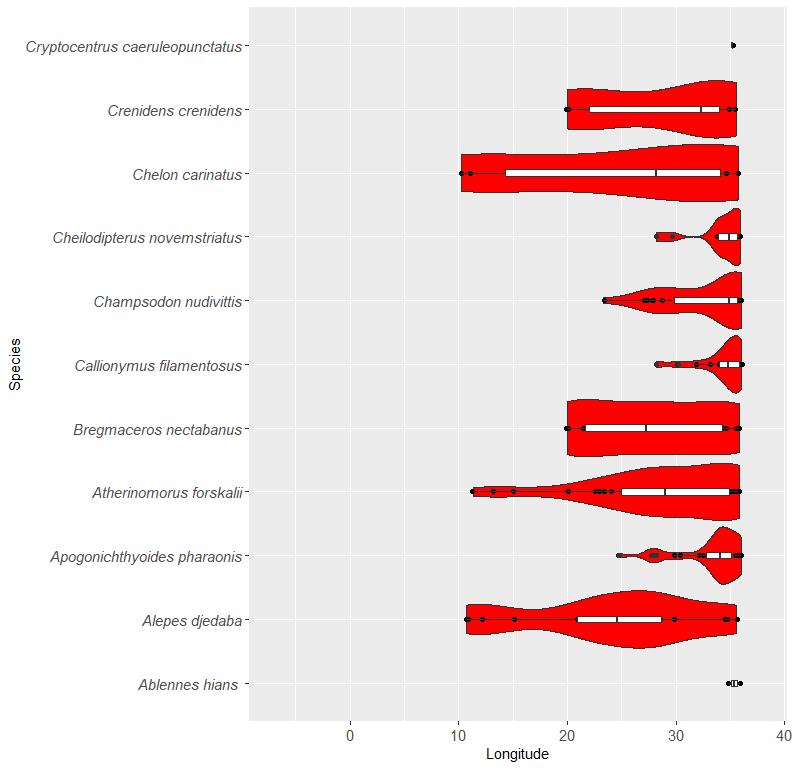


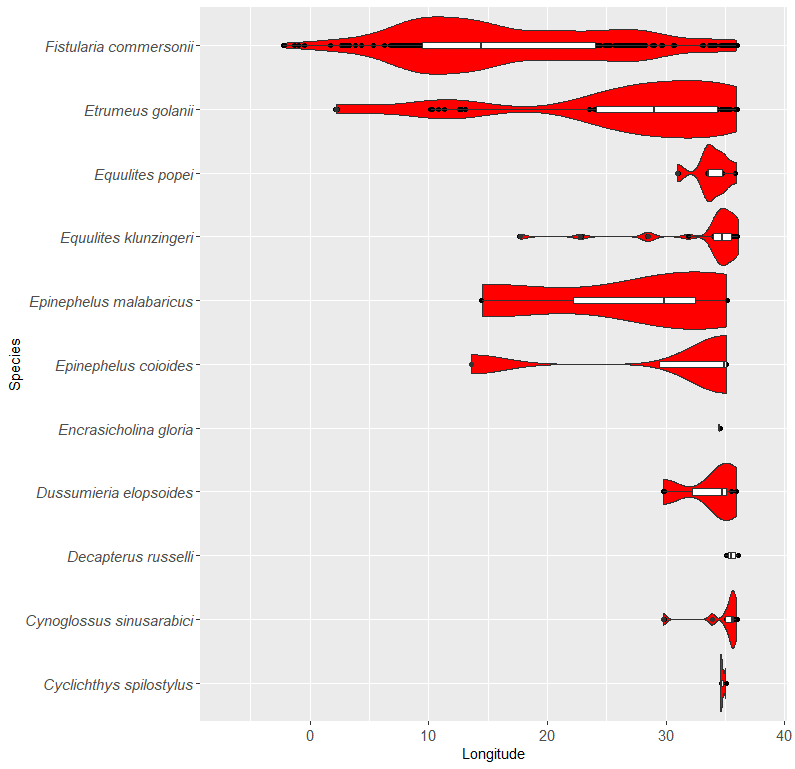


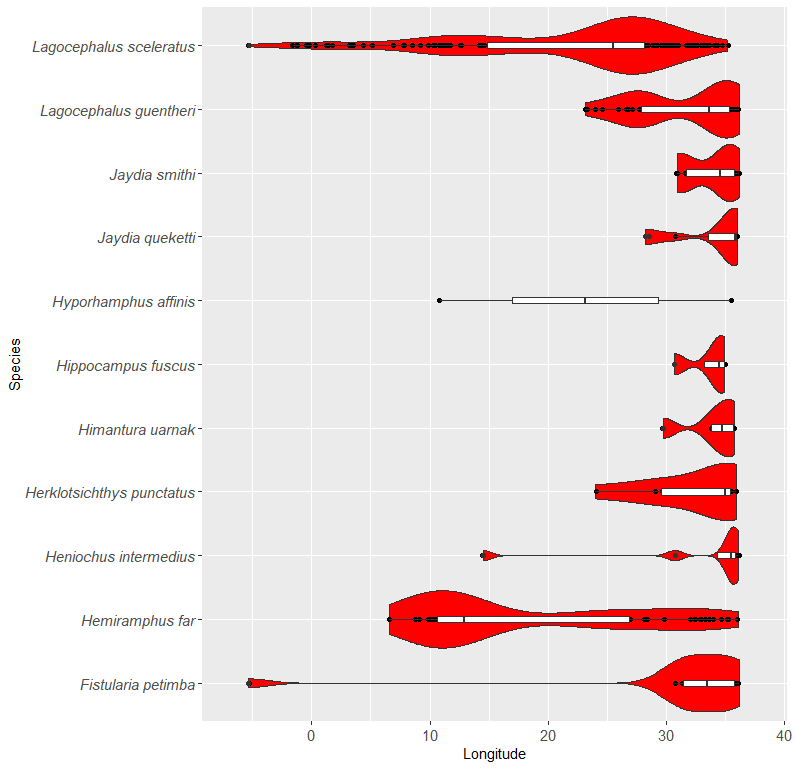


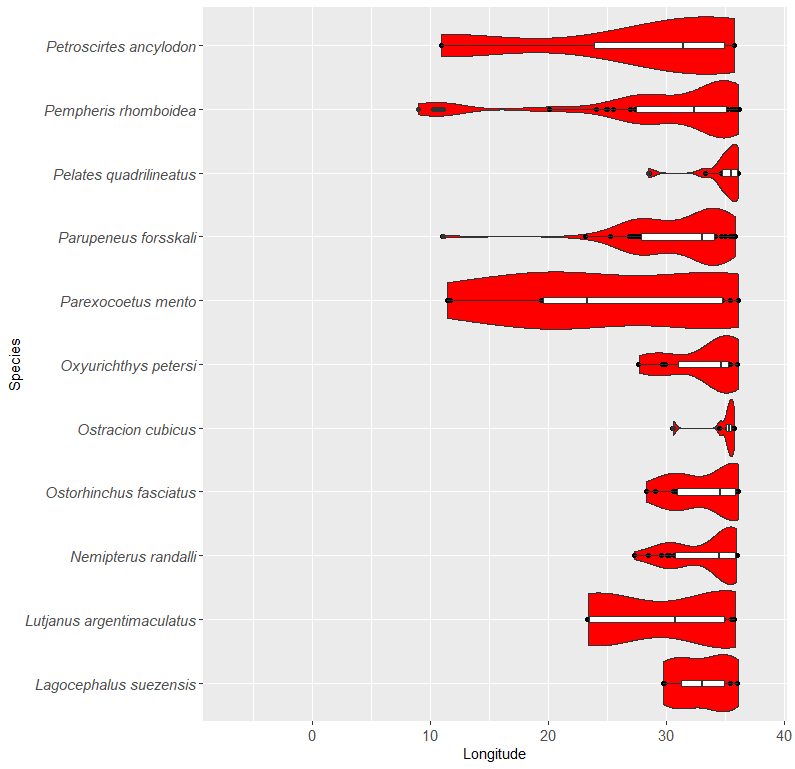


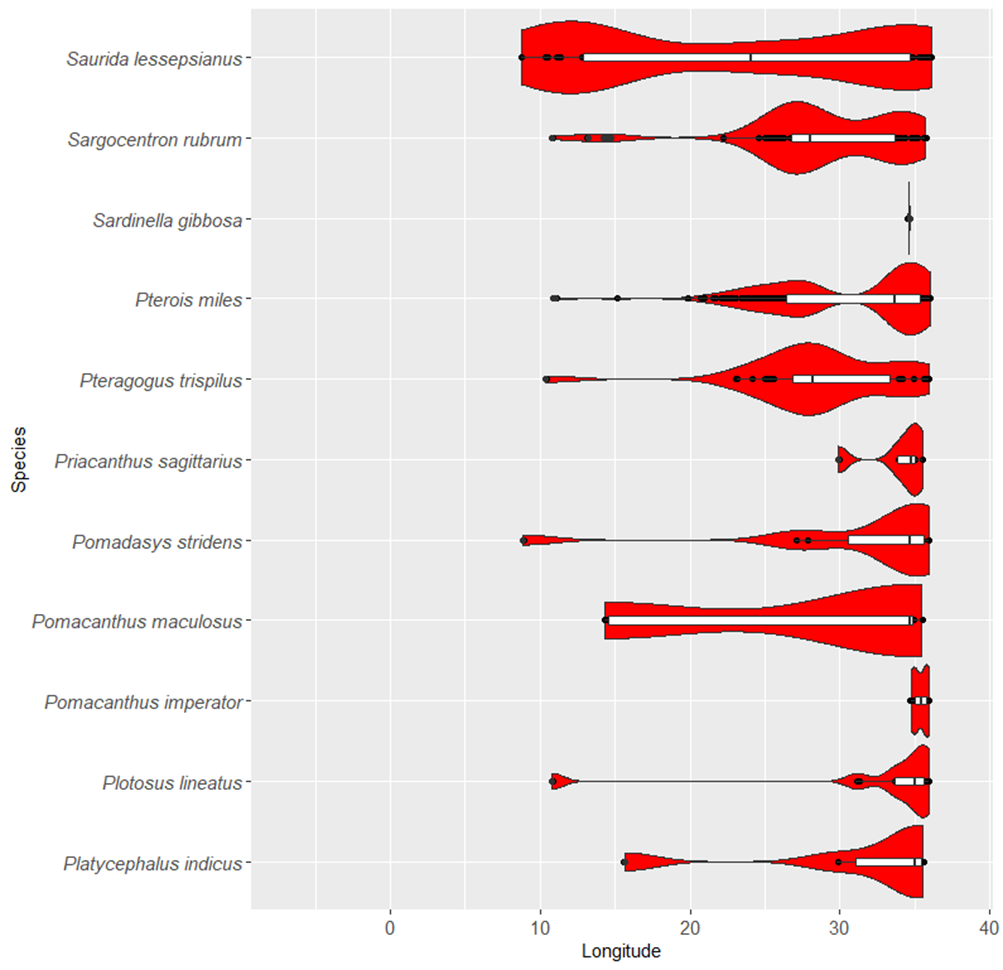


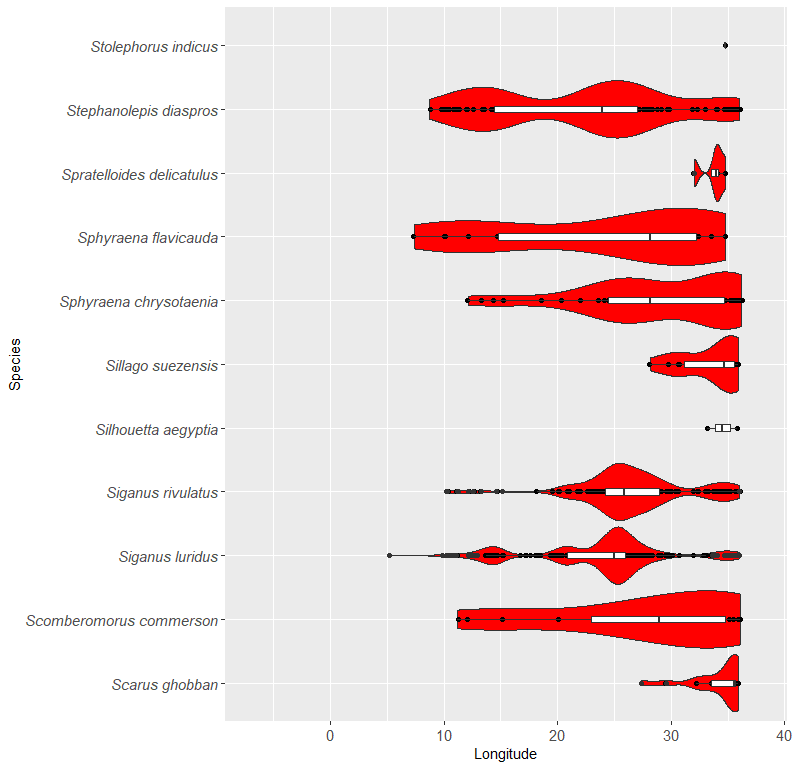


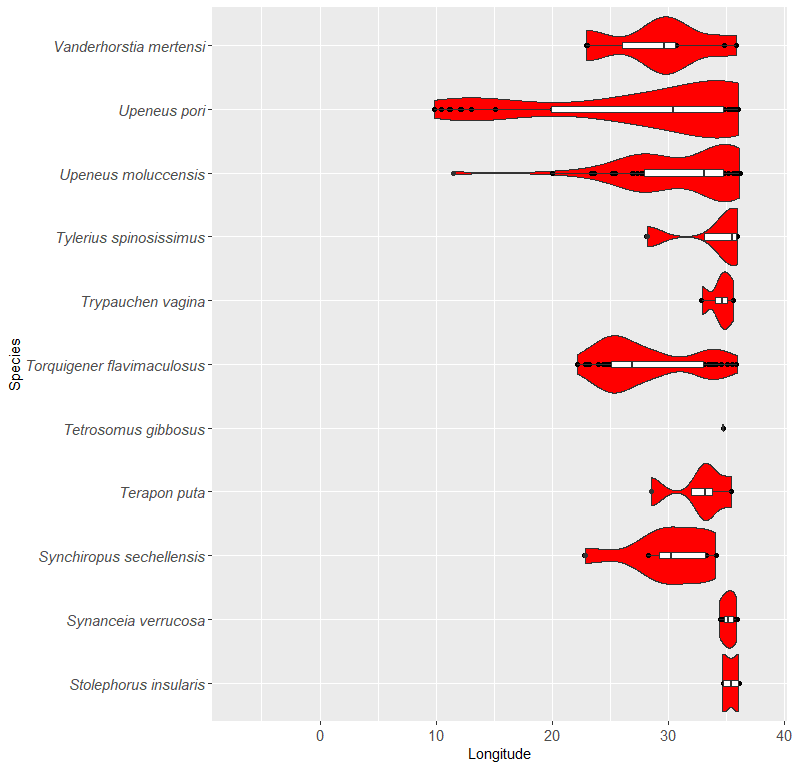


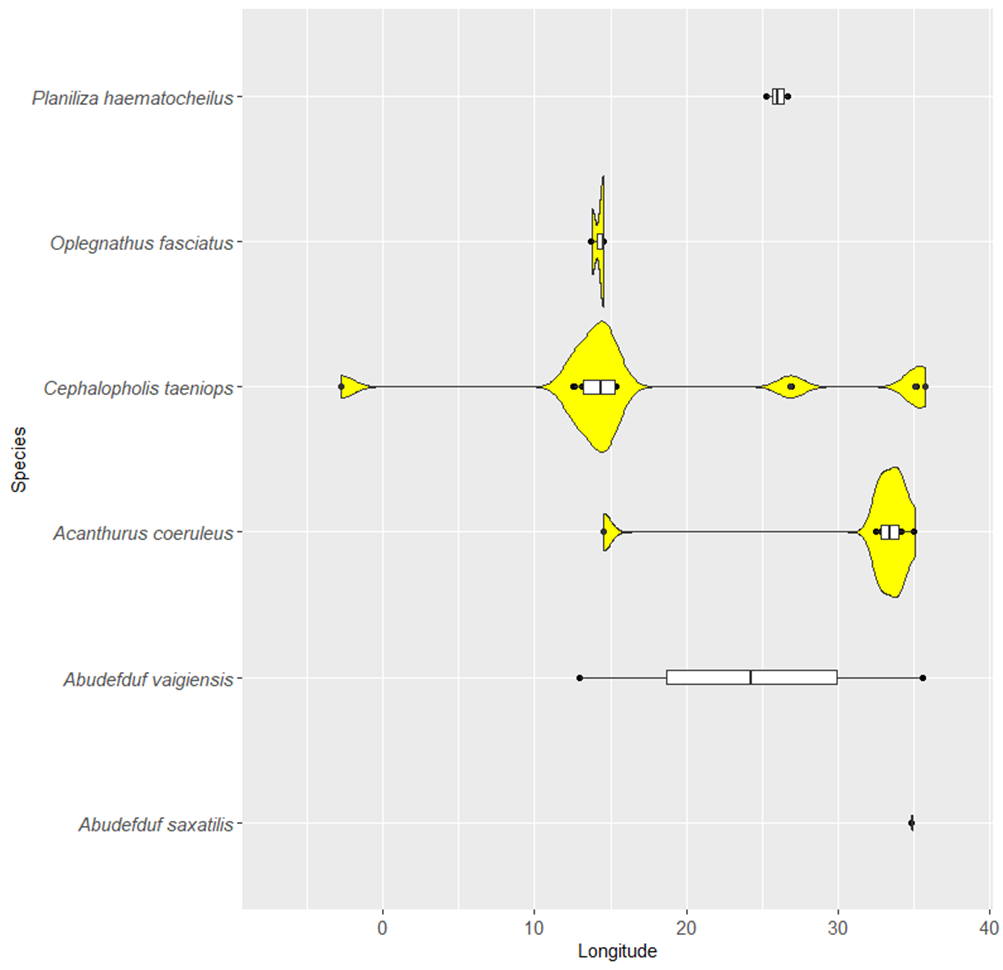


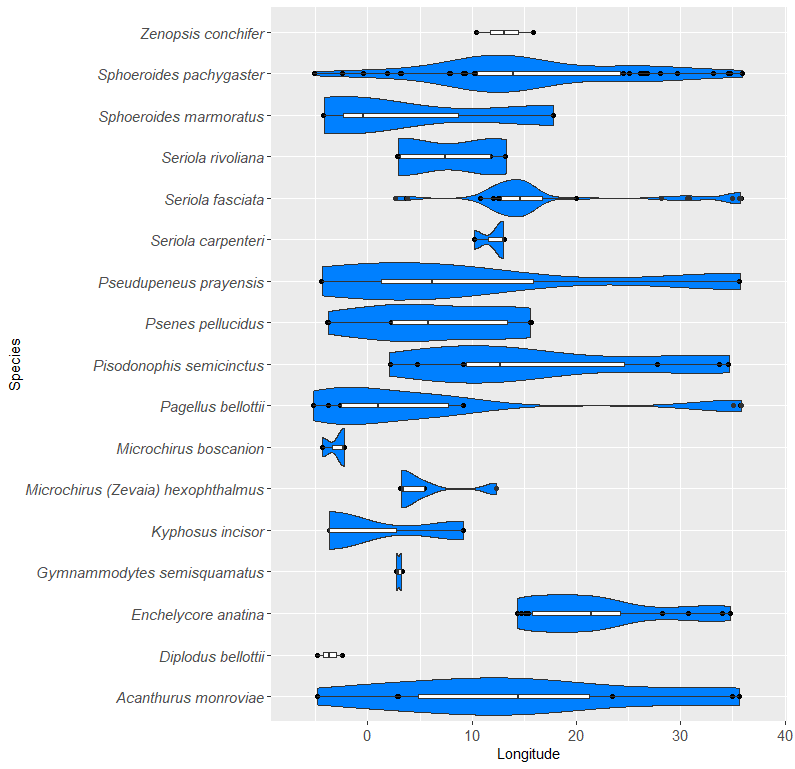

Supplement: Supplementary file 2 — Appendix S2 [file GCB-28-6268-s004.docx]
