# Supplementary material for: Spatio‐temporal dynamics of exotic fish species in the Mediterranean Sea: Over a century of invasion reconstructed
Source: Glob Chang Biol. 2022 Sep 2;28(21):6268–79. doi: 10.1111/gcb.16362 (PMC9826093; doi:10.1111/gcb.16362)
Supplement: Supplementary file 3 — Appendix S3 [file GCB-28-6268-s002.docx]

**SUPPLEMENTARY MATERIALS: Appendix 3**

**Supplementary results on the spatial dynamics of CAN and NRE species**

The spatial dynamics of both CAN and NRE species were first explored and compared through the analysis of the cumulative distribution of species and observations along the longitudinal axis (Fig. S27). According to our data, only 32% of CAN species have been also reported along African coasts further than Egypt. In fact, both the numbers of Lessepsian species and records decrease abruptly westward, with 60% of occurrences observed between 25° and 36° longitudinal degrees (coasts of Egypt, Israel, Lebanon, Syria, Turkey and Greece). The numbers of species and records gradually decrease moving westward to the coasts of Sicily and Tunisia where another abrupt decrease occurs (Fig.S27 a).

Regarding the Natural range expansion species, we observed a severe decrease of the total number of species and observations in correspondence of the Sicily Strait (Fig. S27 b) with few species being able to cross the Strait, such as *Pagellus bellottii* and *Seriola fasciata*.

| 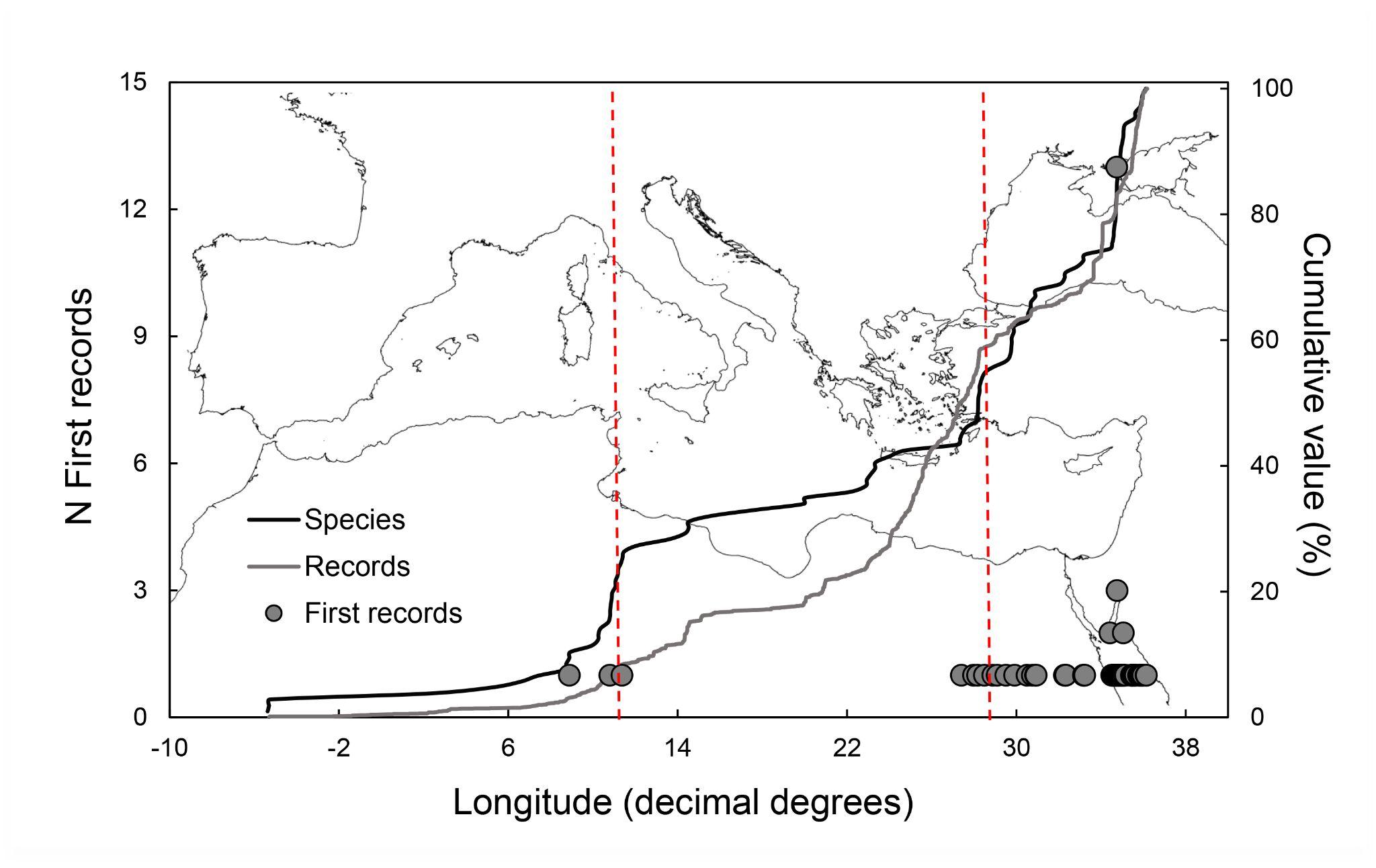 | 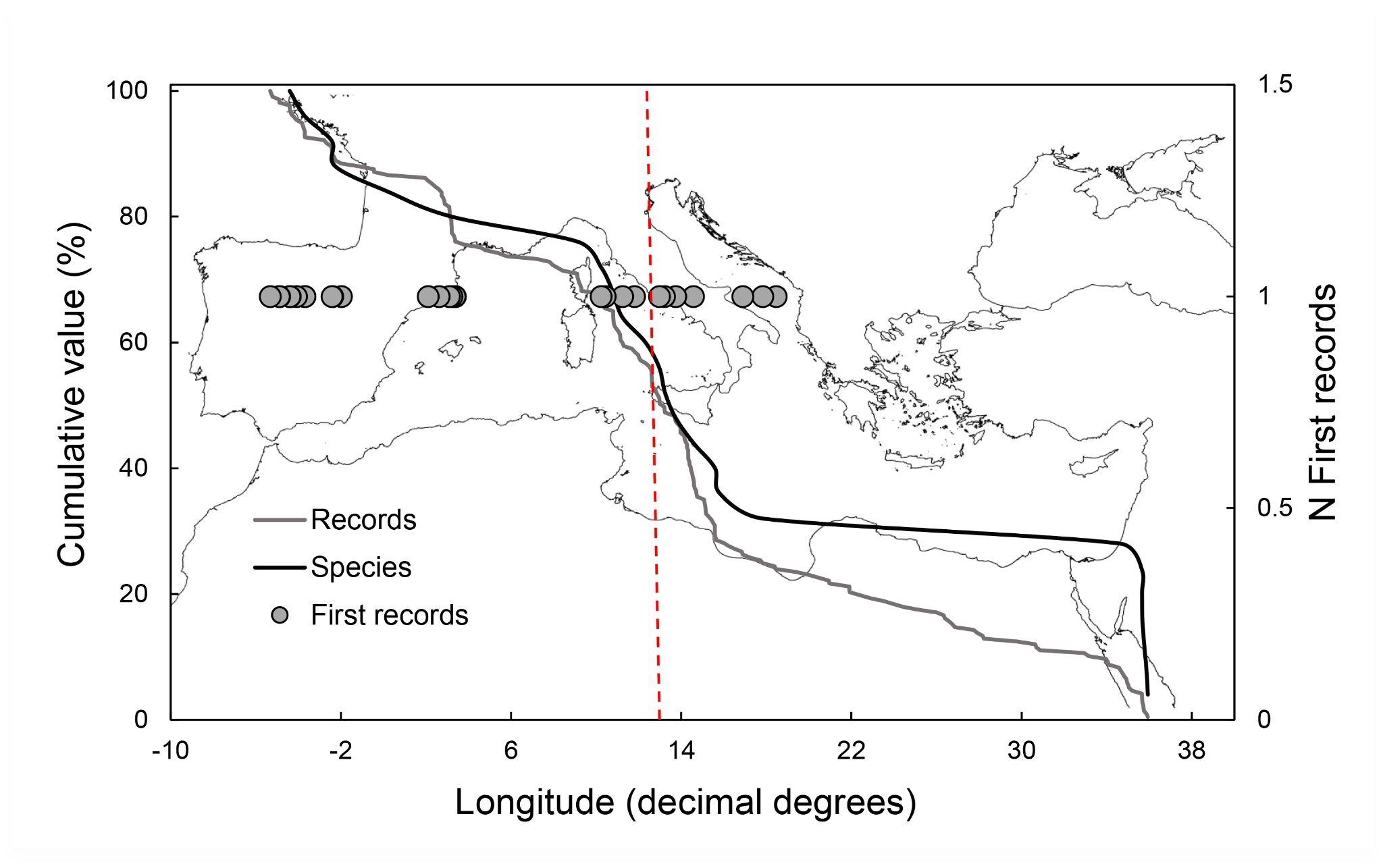 |
| --- | --- |

**Figure S27** Cumulative percentage of total species and occurrences for CAN fish group (left panel) and NRE fish group (right panel) along the longitudinal axis. The red dashed lines represent the breakpoints calculated on the cumulative curves of species accumulation for each fish group.
