# Supplementary material for: Spatio‐temporal dynamics of exotic fish species in the Mediterranean Sea: Over a century of invasion reconstructed
Source: Glob Chang Biol. 2022 Sep 2;28(21):6268–79. doi: 10.1111/gcb.16362 (PMC9826093; doi:10.1111/gcb.16362)
Supplement: Supplementary file 4 — Appendix S4 [file GCB-28-6268-s003.docx]

**Supplementary materials: Appendix 4**

**Boxplots showing the relation between the longitudinal and latitudinal range sizes and number of records for each species.**


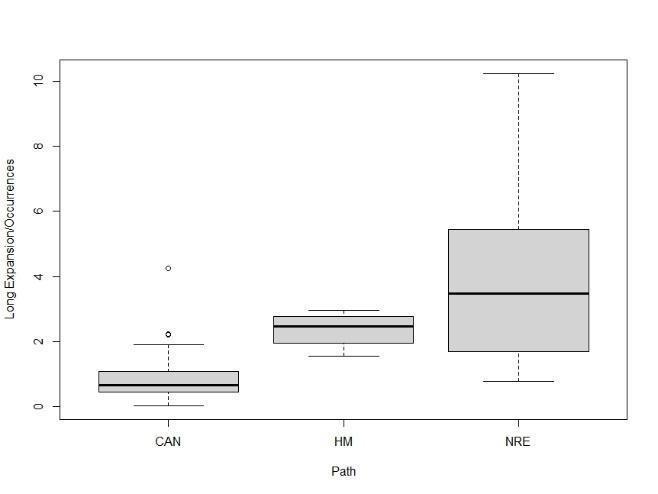

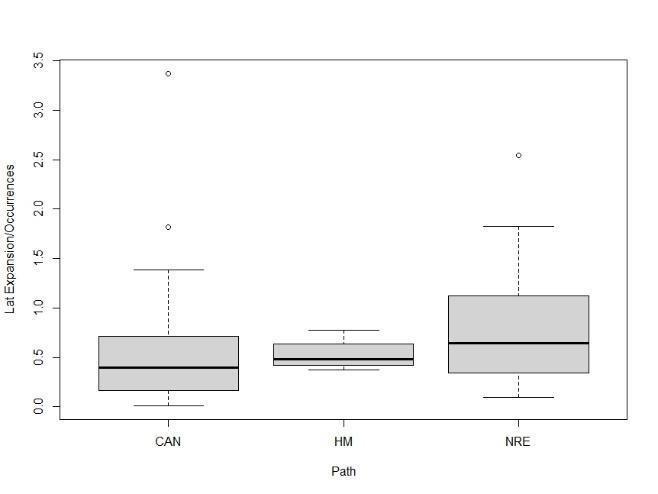


The boxplots compare the Lessepsian (CAN), Natural range expansion (NRE) and Human mediated transport (HM) groups of species on the basis of the relation between the longitudinal (left panel) and latitudinal (right panel) range size and number of records for each species. The resulting value should be intended as a measure of the average distance between two different observations of the same species.
