# Supplementary material for: Spatio‐temporal dynamics of exotic fish species in the Mediterranean Sea: Over a century of invasion reconstructed
Source: Glob Chang Biol. 2022 Sep 2;28(21):6268–79. doi: 10.1111/gcb.16362 (PMC9826093; doi:10.1111/gcb.16362)
Supplement: Supplementary file 5 — Appendix S5 [file GCB-28-6268-s005.docx]

**SUPPLEMENTARY MATERIALS: Appendix 5**

**Supplementary results on the fish spread rate**

Concerning the calculation of the spread rate based on the analysis of species maximum expansion along the longitudinal axis in successive time periods, results were highly correlated with the least-cost method.

The median, minimum and maximum spread rate of each category were: CAN_N_ = 0.69, 0.15, 2.98; CAN_S_ = 0.63, 0.14, 2.70; NRE_N_ = 0.28, 0.13, 1.3; NRE_S_ = 1.25, 0.52, 3.09.


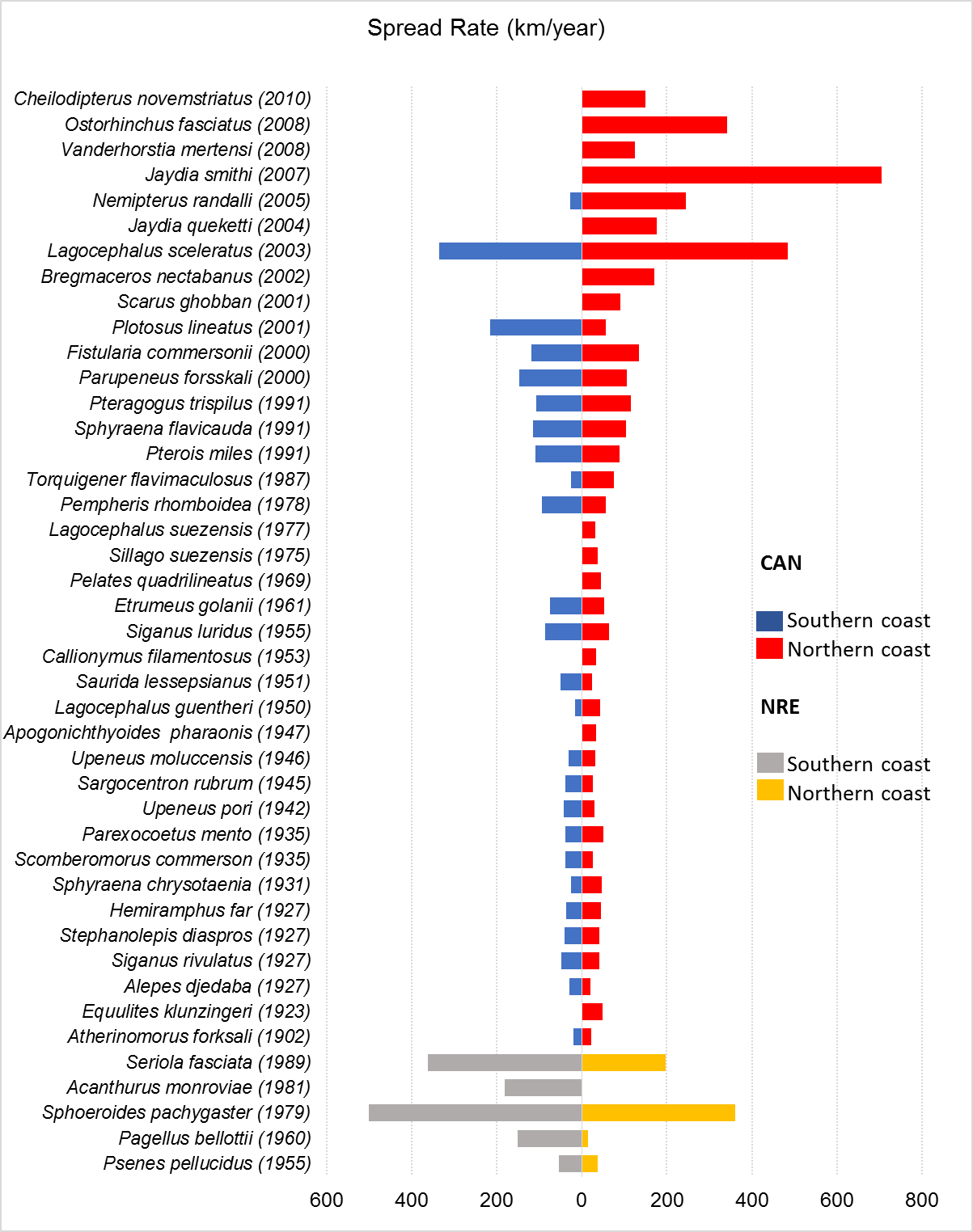


The histogram shows the average spread rate calculated as km/year along the Northern and Southern coast, separately, for the 38 and 5 CAN and NRE species, respectively, with more than 10 records in the Mediterranean Sea. Species are ordered according to the year of the first observation (in brackets), from the most recent species to the oldest ones.


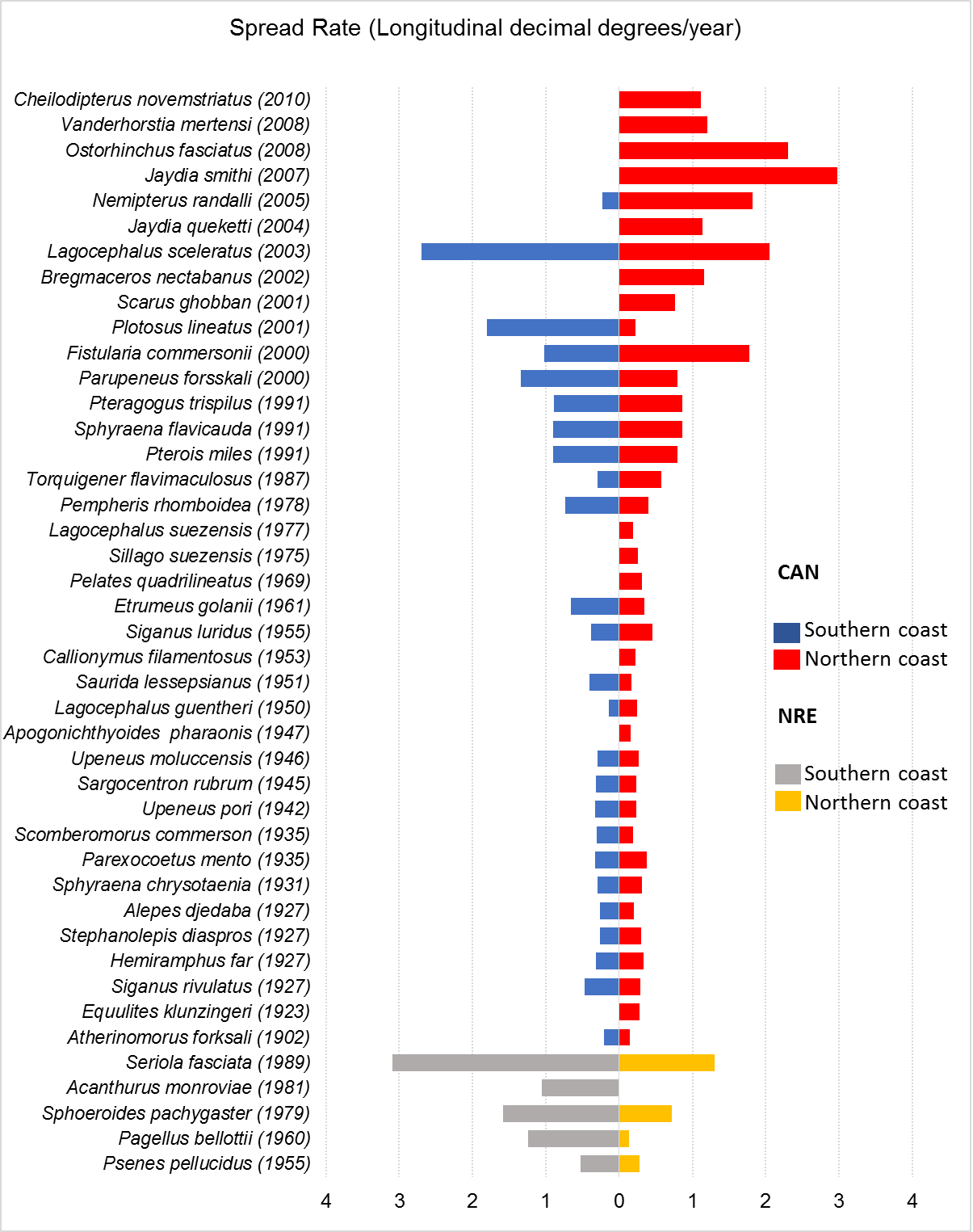


The histogram shows the average spread rate calculated as longitudinal decimal degrees/year along the Northern and Southern coast for the 38 and 5 CAN and NRE species, respectively, with more than 10 records in the Mediterranean Sea. Species are ordered according to the year of the first observation (in brackets), from the most recent species to the oldest ones. Consider that 1 decimal degree corresponds to ca. 85 km at the central latitude of the Mediterranean Sea.
